# Molecular dynamics simulations of cyanine dimers attached to DNA Holliday junctions†

**DOI:** 10.1039/d2ra05045e

**Published:** 2022-10-04

**Authors:** Austin Biaggne, Young C. Kim, Joseph. S. Melinger, William B. Knowlton, Bernard Yurke, Lan Li

**Affiliations:** Micron School of Materials Science and Engineering, Boise State University Boise ID 83725 USA lanli@boisestate.edu; Materials Science and Technology Division, U.S. Naval Research Laboratory Washington DC 20375 USA; Electronics Science and Technology Division, U.S. Naval Research Laboratory Washington DC 20375 USA; Department of Electrical and Computer Engineering, Boise State University Boise ID 83725 USA; Center for Advanced Energy Studies Idaho Falls ID 83401 USA

## Abstract

Dye aggregates and their excitonic properties are of interest for their applications to organic photovoltaics, non-linear optics, and quantum information systems. DNA scaffolding has been shown to be effective at promoting the aggregation of dyes in a controllable manner. Specifically, isolated DNA Holliday junctions have been used to achieve strongly coupled cyanine dye dimers. However, the structural properties of the dimers and the DNA, as well as the role of Holliday junction isomerization are not fully understood. To study the dynamics of cyanine dimers in DNA, molecular dynamics simulations were carried out for adjacent and transverse dimers attached to Holliday junctions in two different isomers. It was found that dyes attached to adjacent strands in the junction exhibit stronger dye-DNA interactions and larger inter-dye separations compared to transversely attached dimers, as well as end-to-end arrangements. Transverse dimers exhibit lower inter-dye separations and more stacked configurations. Furthermore, differences in Holliday junction isomer are analyzed and compared to dye orientations. For transverse dyes exhibiting the smaller inter-dye separations, excitonic couplings were calculated and shown to be in agreement with experiment. Our results suggested that dye attachment locations on DNA Holliday junctions affect dye-DNA interactions, dye dynamics, and resultant dye orientations which can guide the design of DNA-templated cyanine dimers with desired properties.

## Abstract 2

Molecular dynamics simulations reveal dye attachment and DNA Holliday junction isomer effects on dye dimer orientations and excitonic couplings. These simulations can guide synthesis and experiments of dye-DNA structures for excitonic applications.

## Graphical abstract

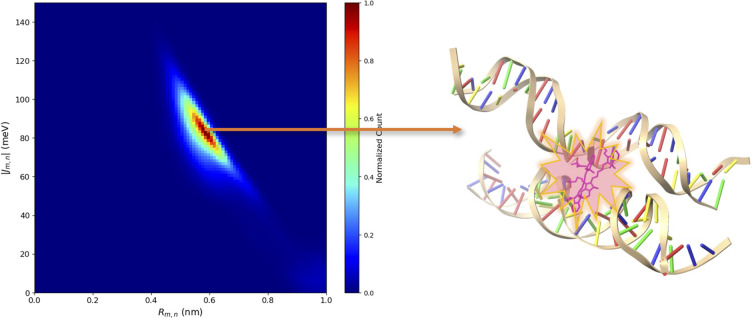

## 1. Introduction

Aggregates of dye molecules play a critical role in numerous applications, including organic photovoltaics,^1,2^ non-linear optics,^3^ and quantum information systems.^4–8^ Central to such applications are delocalized excitations, or excitons, which reside on the dye aggregates to facilitate highly efficient energy transfer. This energy transfer allows for the movement of excitons across excitonic wires,^5,9^ from antennae to reaction centers,^10,11^ or to charge separation interfaces between electron donor and acceptor materials.^12,13^ The functionality of molecular materials employing excitons relies on the properties of the dyes and their aggregates. In the case of quantum information and computing systems, the precise control and knowledge of dye orientations are necessary.

Dyes are known to aggregate in natural systems *via* proteins,^1,10^ as well as artificial systems due to the spontaneous aggregation of dyes in concentrated solutions.^14,15^ However, the spontaneous aggregation of free dyes in solution does not allow for the control of the number of dyes in an aggregate or dye orientations, which are vital to the excitonic properties of aggregates. The dynamics of excitons on dye aggregates depend on both the electronic properties of individual dyes and the orientations of the dyes in the aggregate.^16–19^ Following the work of Scheibe and Jelley,^14,15,20^ considerable effort has been made to study the properties of dye aggregates. Building upon Davydov's initial work on molecular excitons,^21,22^ Kasha described how the orientation of the transition dipole moments of dyes in an aggregate can explain the aggregate's optical properties.^16,17^ In the case of a simple dimer, two energy states are allowed. The stacked configuration of two dyes (called an H-dimer aggregate), yields a blue-shifted absorption spectrum where excitations to the higher energy state, compared to the monomers, are allowed and excitations to the lower energy state are forbidden. An end-to-end orientation (J-dimer aggregate) yields a red-shifted spectrum, where the higher energy transition is forbidden and the lower energy transition is allowed. A third orientation in which the transition dipoles are at 90° to one another (termed oblique) leads to a splitting of the absorption peak (both higher and lower energy transitions are allowed), known as Davydov splitting.^22^ In Kasha's model,^16,17^ the strength of the coupling of the transition dipole moments is quantified by the exciton exchange energy (*J*_m,n_). This term, also known as excitonic hopping parameter,^23^ enables exciton delocalization and depends upon the strength of the constituent monomer transition dipole moment magnitudes, relative transition dipole moment orientations, and inter-dye distances.^16–19^ Thus, the control of dye orientations is crucial to construct excitonic devices.

An emerging method to control the number and orientation of dyes in an aggregate is through the use of DNA scaffolding. The Watson–Crick DNA base pairing allows for the programmable assembly of DNA into complex structures.^24–26^ It has been shown that DNA scaffolds can be effectively used to form dye aggregates by either non-covalent^27–30^ or covalent binding of the dyes to the DNA *via* tethers.^31–39^ In the case of non-covalent binding, there is a lack of precise control of the dye orientations. Conversely, by attaching dyes to DNA using linkers, a variety of aggregates can be formed. For example, dyes attached to DNA duplexes have been shown to exhibit red-shifted absorption spectra^33,38,40,41^ and blue-shifted absorption spectra.^35,39,42^ Recently, multiple dyes (*i.e.*, trimers and tetramers) attached sequentially on a single strand of a duplex have been shown to form H-aggregates.^42^ More complex DNA scaffolds have also been used, such as DNA origami structures^4,8,43–45^ and isolated Holliday junctions.^23,41,46–49^ The latter consists of two bridged DNA duplexes to form a four-arm junction. Cannon *et al.*^46^ and Huff *et al.*^41^ have shown that two dyes attached to DNA Holliday junctions *via* dual tethers can from both J- and H-aggregates, depending on if the dyes are attached to adjacent or transverse strands, respectively. Furthermore, Huff *et al.* have demonstrated that Cy5 dimers attached adjacently to DNA Holliday junctions exhibit heterogeneity in their configurations, indicating that the precise control of the orientations can be improved.^41^ Chowdhury *et al.* have recently shown that, compared to homodimers, heterodimers consisting of a Cy5 and a Cy5.5 dye can change the optical properties of the aggregate by affecting the dye orientations and couplings.^47^

Efforts have been made to study the electronic and structural properties of dyes and dye-DNA constructs using computational modeling. Density functional theory (DFT) and time-dependent (TD-) DFT have been employed to determine the effects of substituents on the ground and excited state properties of dye monomers.^50–52^ However, DFT and TD-DFT are limited to only a few hundred atoms, and so are not suited for the modeling of dyes attached to DNA scaffolds. Molecular dynamics (MD) simulations have been employed to study the orientations of dyes attached to DNA structures.^33,44,45,53–56^ MD simulations of dye dimers^33,53^ and trimers^53^ attached to DNA duplexes have been shown to yield dye orientations in agreement with experimental observations. MD simulations of dyes attached to larger DNA complexes, such as bundles and origami,^44,45^ have also been shown to yield dye orientations and dynamics in agreement with experiment. Furthermore, MD simulations of Holliday junctions have also been performed.^44,57–59^ Mathur *et al.* has showed that MD simulations of dyes attached to the Holliday junctions of large DNA bundles can yield dye orientations in agreement with experimental fluorescence data.^44^ Wheatley *et al.* found that isolated, four-arm Holliday junctions initialized in an open conformation stabilize in a stacked configuration at both high and low salt concentrations, indicating that MD is able to capture the conformational transitions of the Holliday junction.^57^ Simmons *et al.* successfully used MD simulations to explore ion binding sites in isolated Holliday junctions.^58^ Adendorff *et al.* utilized MD to study the structure and dynamics of four-arm isolated Holliday junctions to understand the impact of base sequences on the junction structure.^59^ However, the role of the DNA Holliday junction conformations and the interactions between the DNA and dyes that affect dye orientations and resultant excitonic properties are not fully understood.

In this work, MD simulations of Cy5 and Cy5.5 dyes attached to Holliday junctions were performed to understand dye orientations, dye interactions with DNA, and how they influence the homodimers and heterodimers, consisting of Cy5 and Cy5.5 dyes, in comparison with experimental measurements in support of previous studies.^41,46,47^ To obtain dye orientations from experimental measurements, an in-house program developed based on the theoretical model of Kühn, Renger, and May (KRM) was used. A full description of the model is given in ref. 23, 40 and 46, but in short, the model accounts for the dominant vibrational mode of each molecule in the aggregate non-perturbatively, producing theoretical absorbance and circular dichroism (CD) spectra of various dye configurations. The produced theoretical spectra are then compared to the experimental spectra until a best fit is found. An extended dipole approximation is used to determine *J*_m,n_ between the two dyes in the resultant orientation.^19^ In the past few years, our group has successfully used the KRM model to study the aggregates of dyes covalently bound to DNA scaffolds.^23,38,40,42,46–49,60^ Specifically, the four-arm isolated Holliday junction has been shown to be useful to produce strongly coupled dimers.^23,41,46,47,49^ MD is an alternative option to study the dye–dye and dye–DNA interactions to provide further insight into the systems of interest.

To study the intermolecular interactions of dyes attached to isolated DNA Holliday junctions, we first performed μs long simulations of Cy5 homodimers, Cy5.5 homodimers, and Cy5–Cy5.5 heterodimers attached to DNA Holliday junctions in either adjacent or transverse configurations. The MD trajectories were then used to compute the dye orientation factors (*κ*_m,n_), dye center-to-center distances (*R*_m,n_), *J*_m,n_, and contact maps with the surrounding DNA. The dye orientation results were also compared to the results obtained from the modeling of experimental excited state measurements of dimers on DNA Holliday junctions.^41,47^

## 2. Methods

### 2.1 Molecular dynamics simulations

MD simulations were performed to determine the orientations of the dye dimers and interactions of the dyes with the DNA. The OL15 forcefield,^58,61,62^ which had been tested for base flipping in DNA and TNA duplexes,^63,64^ was used with non-bonded modifications for the DNA phosphate-ion interactions.^65^ Similar to previous studies,^45,53^ the general Amber forcefield (also known as GAFF) was used for dye bonded and van der Waals parameters^66^ that were generated using the ANTECHAMBER software.^67^ The dye atomic point charges were derived using the restrained electrostatic potential fit method^68^ to reproduce the electrostatic potential calculated at the HF/6-31G* theory level. The dye-DNA structures were solvated in TIP3P water and neutralized with Mg^2+^ (ref. 69 and 70) and Cl^−^ (ref. 71) ions, with 15 mM MgCl_2_ excess salt concentration added to match experimental conditions.^41,47^ A truncated octahedron box was used, ensuring 1.2 nm separation between dye-DNA and box edges. Neighbor-searching was utilized with a cutoff of 1.2 nm and van der Waals interactions were limited to 1.2 nm with the forces smoothly switched to zero starting at 1.0 nm, like similar studies.^72,73^ Long range dispersion corrections were applied for energy and pressure. The particle-mesh Ewald method^74,75^ was utilized with a real-space coulomb cutoff of 1.2 nm. Bonds to hydrogen atoms were constrained *via* the LINCS algorithm^76^ and a timestep of 2 fs was used. All simulations were performed using the GROMACS 2020.3 software package.^77^

**Fig. 1:**
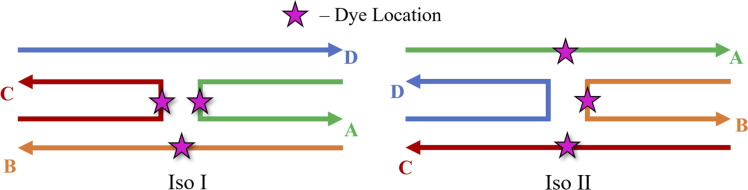
Schematics of the Holliday junction isomers considered for MD simulations from Huff *et al.*^41^ and Chowdhury *et al.*^47^ The stars represent possible dye locations depending on the system – for transverse dimers, dyes are attached on the A and C strands and for adjacent dimers, dyes are attached to the A and B strands.

**Fig. 2:**
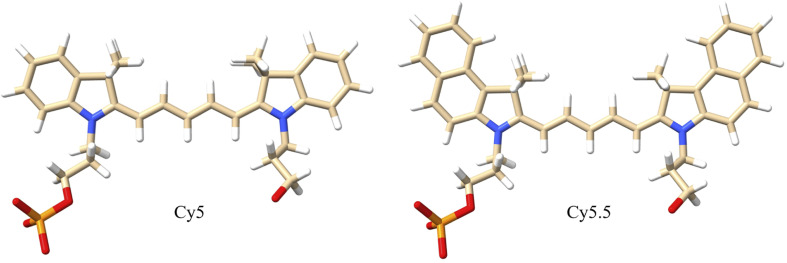
Molecular structures of Cy5 and Cy5.5 dyes with dual phosphoramidite linkers that attach to the 5′ and 3′ ends of the DNA backbone. The dyes consist of pentamethine bridges flanked by two dimethyl indolenine rings. The Cy5.5 dye contains two extra aryl rings that extend the π-conjugation. White atoms are hydrogen, tan atoms are carbon, blue atoms are nitrogen, red atoms are oxygen, and orange atoms are phosphorus.

It is theorized that isolated Holliday junctions exist predominantly in stacked configurations in 15 mM MgCl_2_ solution,^41^ labeled as Iso I and Iso II in Fig. 1. Because of this, the Holliday junctions for the MD simulations were built in both the Iso I and Iso II configurations. Using the Nanoengineer-1 software,^78^ the DNA Holliday junctions were first constructed in both configurations and energy-minimized. Then, the dyes were added to construct the initial DNA–dye structures using the UCSF ChimeraX software.^79^ Following the work done by Huff *et al.*^41^ and Chowdhury *et al.*,^47^ two types of dimers were considered – adjacent and transverse. Adjacent dimers refer to dyes residing on adjacent DNA strands (*i.e.*, strands A and B in Fig. 1), whereas transverse dimers refer to dyes residing on transverse DNA strands (strands A and C). Furthermore, homodimers and heterodimers consisting of Cy5 and Cy5.5 dyes were used. The dyes were placed at positions shown in Fig. 1, and attached to the DNA backbone *via* dual phosphoramidite linkers, as shown in Fig. 2. In total, twelve systems were simulated to encompass all combinations of dye types, isomers, and dimer types. DNA Holliday junction sequences and dye attachment schemes used in the present study are the same as the ones used by Huff *et al.*^41^ and Chowdhury *et al.*^47^

To prepare the dye–DNA systems for production MD simulations, the total energy of the systems was first minimized using the steepest descent method for 5000 steps. Following energy minimization, in order to equilibrate the water and ions, two 1 ns equilibration steps were performed with restraints of 1000 kJ mol^−1^ nm^−2^ and 500 kJ mol^−1^ nm^−2^ on the heavy dye-DNA atoms, respectively, keeping the number of atoms, volume, and temperature constant (*i.e.*, the NVT ensemble). After that, a third equilibration step of 1 ns was performed without restraints. Production MD simulations were run for 1 μs, keeping the number of atoms, pressure, and temperature constant (*i.e.*, the NPT ensemble), and it was ensured that the pressure and temperature were well converged prior to data collection. All simulations were run at 300 K and 1 atm. The velocity-rescale thermostat^80^ was used with a coupling time of 0.1 ps. The Parrinello–Rahman^81^ barostat was used with a coupling time of 1.0 ps. Coordinates were written every 10 ps, and the first 100 ns of the final production simulations was not used for data analysis. For each system, three 1 μs simulation trials were run with different initial velocities for enhanced numerical sampling.

### 2.2 Analysis of dye orientation and dye–DNA interaction

To quantify the orientations of the dye dimers, *κ*_m,n_ of the two dyes was calculated using the equation^33^

1 *κ*_m,n_ = *

<svg xmlns="http://www.w3.org/2000/svg" version="1.0" width="12.000000pt" height="16.000000pt" viewBox="0 0 12.000000 16.000000" preserveAspectRatio="xMidYMid meet"><metadata>
Created by potrace 1.16, written by Peter Selinger 2001-2019
</metadata><g transform="translate(1.000000,15.000000) scale(0.012500,-0.012500)" fill="currentColor" stroke="none"><path d="M480 1080 l0 -40 -40 0 -40 0 0 -40 0 -40 -40 0 -40 0 0 -40 0 -40 40 0 40 0 0 40 0 40 40 0 40 0 0 40 0 40 40 0 40 0 0 -40 0 -40 40 0 40 0 0 -40 0 -40 40 0 40 0 0 40 0 40 -40 0 -40 0 0 40 0 40 -40 0 -40 0 0 40 0 40 -40 0 -40 0 0 -40z M320 720 l0 -80 -40 0 -40 0 0 -120 0 -120 -40 0 -40 0 0 -120 0 -120 -40 0 -40 0 0 -80 0 -80 40 0 40 0 0 80 0 80 40 0 40 0 0 40 0 40 120 0 120 0 0 40 0 40 40 0 40 0 0 -40 0 -40 40 0 40 0 0 40 0 40 40 0 40 0 0 40 0 40 -40 0 -40 0 0 -40 0 -40 -40 0 -40 0 0 80 0 80 40 0 40 0 0 120 0 120 40 0 40 0 0 40 0 40 -40 0 -40 0 0 -40 0 -40 -40 0 -40 0 0 -120 0 -120 -40 0 -40 0 0 -80 0 -80 -120 0 -120 0 0 40 0 40 40 0 40 0 0 120 0 120 40 0 40 0 0 80 0 80 -40 0 -40 0 0 -80z"/></g></svg>

*_m_·**_n_ − 3(*R̂*_m,n_·**_m_)(*R̂*_m,n_·**_n_)

where **_i_ is the unit transition dipole moment vector pointing along the long axis of dye m or n and *R̂*_m,n_ is the unit vector between the centers of the transition dipole moment vectors of dyes m and n. A value of |*κ*_m,n_| between 0 and 1 indicates two dyes are predominantly stacked and a |*κ*_m,n_| of greater than 1 means the dyes are predominantly end-to-end.

For comparison, with experimental studies of the excited state properties of dimers attached to DNA Holliday junctions, *J*_m,n_ values were calculated using the extended dipole approximation:^19^2
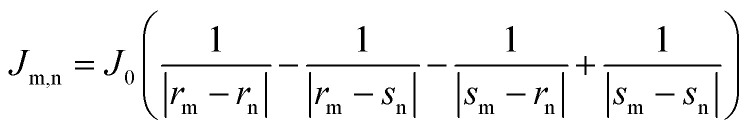
where *r*_i_ and *s*_i_ correspond to the two ends of dye m or n along the long axis. The prefactor term *J*_0_ was calculated as^19^3
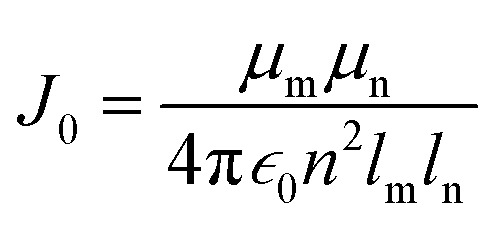
where *μ*_i_ is the transition dipole moment magnitude of dye m or n, *∈*_0_ is vacuum permittivity constant, *n* is the refractive index of water, and *l*_i_ is the length of dye m or n such that *l*_i_ = |*r*_i_ − *s*_i_|. For the calculations of *J*_m,n_, our previous TD-DFT results for *μ*_Cy5_ = 15.35 D and *μ*_Cy5.5_ = 15.57 D (ref. 56) were used.

The dye orientations, quantified using *κ*_m,n_ and *R*_m,n_, were calculated every 100 ps for each MD simulation. The results for each MD trial were combined into one plot for comparison. To clarity, the adjacent and transverse Cy5 homodimers are denoted as A_Cy5_B_Cy5_ and A_Cy5_C_Cy5_, respectively. The Cy5.5 homodimers and Cy5–Cy5.5 heterodimers are denoted similarly. Dye orientations and resultant *J*_m,n_ from the MD simulations were compared to experimental data from Chowdhury *et al.*^47^ in which dye orientations and *J*_m,n_ were extracted from steady-state absorption and CD spectra using the KRM model.

Contact maps of the dyes and DNA bases were generated to quantify how the dyes would interact with the surrounding DNA environment. Each dye and the six center bases of each DNA strand were considered for contact calculations, resulting in 26 groups (or residues). For each DNA residue pair, the distance between the centers of mass of the two constituent residues was calculated. For residue pairs involving dyes, the whole dye center of mass and the ends of the dye (*r*_i_ and *s*_i_ locations) were used for contact calculations to account for the anisotropic structure of the dye. Two residues were considered to be in contact if the shortest distance between their centers of mass (or ends if the residues were dyes) was within 1.2 nm (the same as the simulation short range interaction cut-off). Contacts were calculated every 500 ps for the three simulation trials.

## 3. Results

### 3.1 Adjacent dimers

**Fig. 3:**
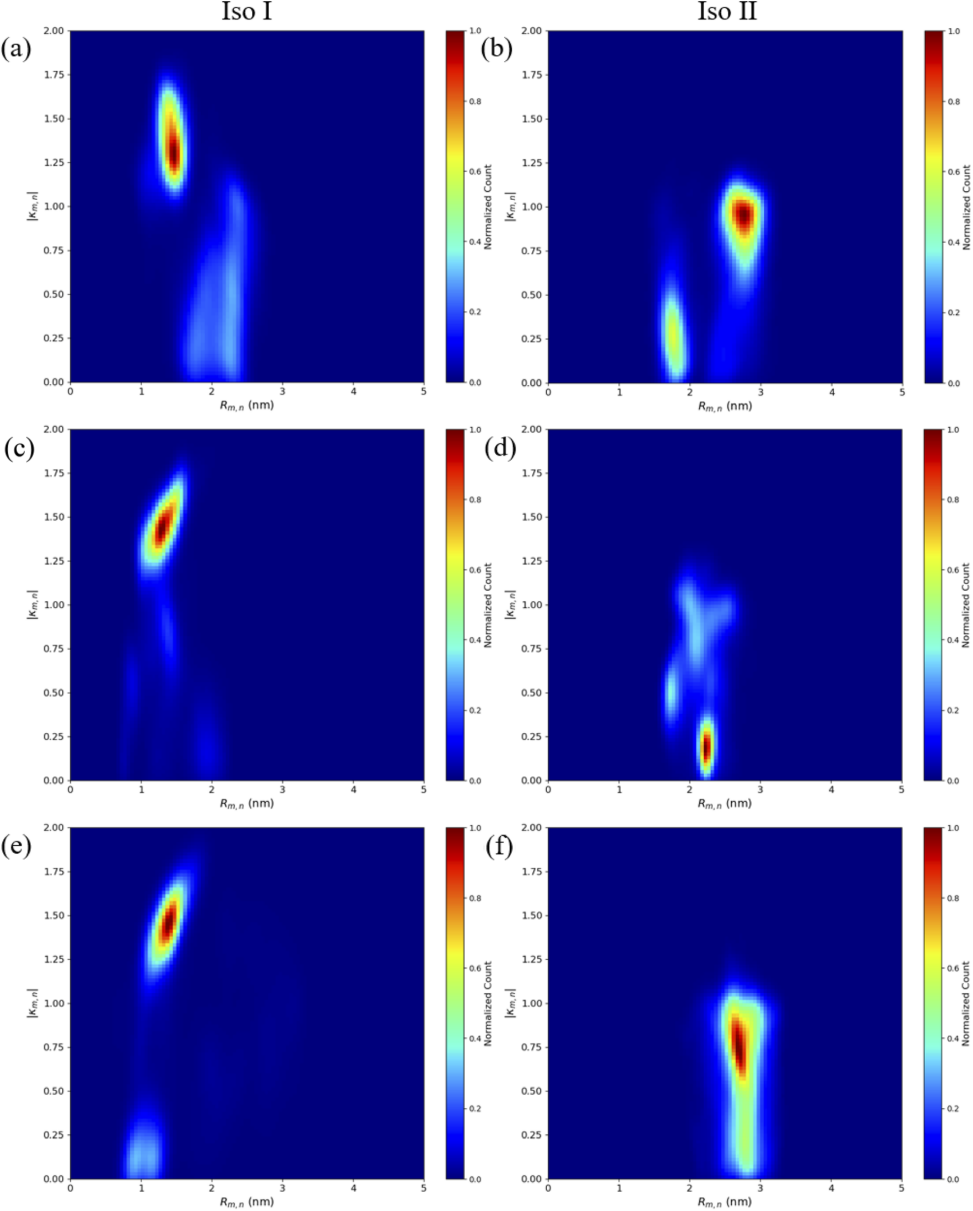
Heatmap plots of |*κ*_m,n_| calculated using eqn (1)*versus R*_m,n_ for (a) A_Cy5_B_Cy5_ Iso I, (b) A_Cy5_B_Cy5_ Iso II, (c) A_Cy5.5_B_Cy5.5_ Iso I, (d) A_Cy5.5_B_Cy5.5_ Iso II, (e) A_Cy5_B_Cy5.5_ Iso I, and (f) A_Cy5_B_Cy5.5_ Iso II.

**Fig. 4:**
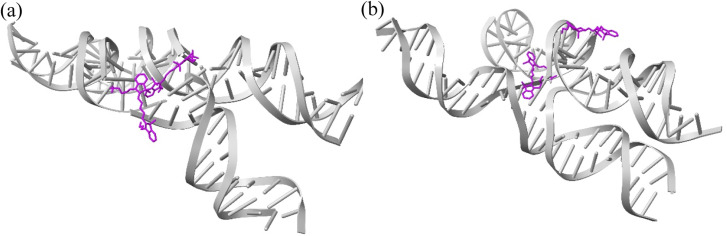
Representative snapshots of A_Cy5_B_Cy5_ Iso I showing the dye orientations when the dyes are (a) in a closely spaced J-like dimer (*κ*_m,n_ ≈ 1.6) and (b) a separated, H-like arrangement (*κ*_m,n_ ≈ 0.2).

The MD results for the adjacent dimers in both Iso I and Iso II Holliday junction conformations are presented in Fig. 3 and 4. Fig. 3 shows heatmap plots of |*κ*_m,n_|, calculated using eqn (1), *versus R*_m,n_. The heatmaps represent 2-dimensional histograms consisting of “binned” data (*e.g.*, |*κ*_m,n_| and *R*_m,n_), where the color scale represents the normalized count in each bin. Data from the three separate, 1 μs MD simulations (*i.e.*, trial 1, trial 2, and trial 3) are combined in each heatmap. Plots of the adjacent dimer orientations as a function of time for the three 1 μs MD simulation trials are given in Fig. S2.† As shown in Fig. S2,† the calculated *R*_m,n_ and *κ*_m,n_ of the three MD simulation trials are comparable over the 900 ns trajectories, indicating that the sampled dye configurations are consistent with each other. One exception is the adjacent Cy5 homodimer – for both Iso I and Iso II, the trajectories shown in Fig. S2a and b† indicate that the dyes may be exploring different local minima. One reason for this, compared to the Cy5.5 homodimer and the Cy5/Cy5.5 heterodimer, may be due to the smaller size of the Cy5 dyes in the Cy5 homodimer. The smaller size of the Cy5 dyes may allow for additional configurations when interacting with the DNA. This may be addressed by performing more simulations, longer simulations, or different sampling techniques, which are outside the scope of this paper. Furthermore, *R*_m,n_ for all simulations converge to stable values after approximately 100 ns, indicating that the MD simulations have achieved stable dye distances. For additional comparison between the three trials, the average values of *R*_m,n_ and *κ*_m,n_ (as well as *J*_m,n_ for A_Cy5_C_Cy5_, A_Cy5.5_C_Cy5.5_, and A_Cy5_C_Cy5.5_ Iso I) are provided in Table S2.† Overall, the average values are largely consistent across the three trials for each systems.

As can be seen in Fig. 3a and b, the A_Cy5_B_Cy5_ system adopts four dominant configurations. A |*κ*_m,n_| between 0 and 1 indicates that the dyes are stacked and correspond to a H-like orientations while a |*κ*_m,n|_ greater than 1 indicates that the dyes are end-to-end and thus correspond to a J-like orientation. For Iso I Holliday junction configurations, there are two peaks in the heatmap plot (Fig. 3a). The less populated peak at around *R*_m,n_ = 2.2 nm and |*κ*_m,n_| ≈ 0.3–0.75 corresponds to the dyes being in an H-like orientation (based on the value of |*κ*_m,n_|) but not considered to be in a dimer due to the large inter-dye distance. Conversely, the dominant peak in Fig. 3a corresponds to a smaller *R*_m,n_ ≈ 1.4 nm and |*κ*_m,n_| ≈ 1.25–1.7, which correlates to a slightly oblique J-like dimer. Examining Fig. 3b, it can be seen that the change in Holliday junction isomer results in different dye orientations and distances. The two main peaks correspond to H-like dye arrangements with some obliqueness at roughly *R*_m,n_ ≈ 1.8 nm and *R* ≈ 2.9 nm, respectively, indicating the dyes do not form closely spaced dimers. From Fig. 3c–f, it can be seen that the A_Cy5.5_B_Cy5.5_ and A_Cy5_B_Cy5.5_ dimers follow similar trends with the Iso I Holliday junction configurations having large peaks that correspond to J-like dimers at *R*_m,n_ and |*κ*_m,n_| values comparable to that of the A_Cy5_B_Cy5_ Iso I dimers. Furthermore, the A_Cy5.5_B_Cy5.5_ and A_Cy5_B_Cy5.5_ dimers on the Iso II Holliday junctions adopt H-like stacking arrangements at around *R*_m,n_ ≈ 2.2 nm and *R*_m,n_ ≈ 2.7 nm, respectively, slightly larger than that of the A_Cy5_B_Cy5_ Iso II system. Snapshots of A_Cy5_B_Cy5_ are shown in Fig. 4 to highlight the two dye orientations (J-like and H-like) observed in the MD simulations. Additional snapshots corresponding to the heatmaps in Fig. 3 are shown in Fig. S5 and S6.† As can be seen in Fig. S5,† the Iso I configurations yield the end-to-end dye dimer (*i.e.*, J-like).

**Fig. 5:**
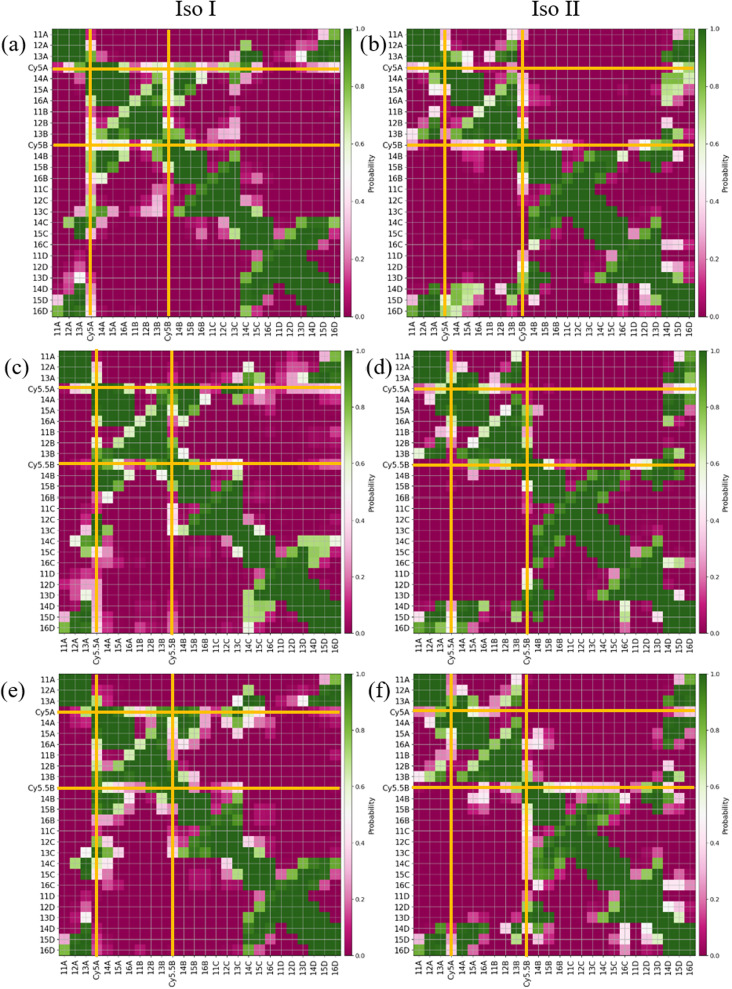
Contact maps for (a) A_Cy5_B_Cy5_ Iso I, (b) A_Cy5_B_Cy5_ Iso II, (c) A_Cy5.5_B_Cy5.5_ Iso I, (d) A_Cy5.5_B_Cy5.5_ Iso II, (e) A_Cy5_B_Cy5.5_ Iso I, and (f) A_Cy5_B_Cy5.5_ Iso II. The *x*- and *y*-axes correspond to the dye or base residue names. Two residues are considered to be in contact if they are within 1.2 nm. Dye contacts with all other residues are indicated in the plots with bold orange lines.

Contact maps between the dyes and DNA bases were calculated from all three simulation trials to elucidate how the dyes interact with the nearby DNA environment. Fig. 5 shows contact maps for the adjacent dimers in both Iso I (Fig. 5a, c and e) and Iso II (Fig. 5b, d and f) Holliday junction configurations. Contact maps were generated for simulation trials 1, 2, and 3, and the bases and/or dyes (collectively termed residues) are said to be in contact if their centers of mass (or ends if the residues are dyes) are within 1.2 nm (the short-range interaction cut-off set for the MD simulation). The diagonal of the contact maps correspond to residues in contact with themselves (*i.e.*, a probability of 1). The axes on Fig. 5 show the residue names of either the DNA bases or dyes. For DNA bases, the residue name consists of the residue number and strand the base belongs to (A, B, C, or D). Each DNA strand consists of 26 bases, so base numbers 11–16 correspond to the bases near the center of the Holliday junction. The dye residue names consist of the dye type (Cy5 or Cy5.5) and the DNA strand the dye is attached to, in regard to Fig. 1. The bins in the contact maps, centered at the intersections of each residue, are color-coded to show the probability of those two residues to be within 1.2 nm of each other (*i.e.*, in contact).

Fig. 5a and b show the contact maps for A_Cy5_B_Cy5_ Iso I and Iso II, respectively. It can be seen that the two dyes are in contact with a probability of around 0.2, which coincides with the two peaks shown in Fig. 3a since the cut-off radius for the contact maps is 1.2 nm. For both Iso I and Iso II, there are peaks present corresponding to the dyes in contact with DNA bases near the center of the Holliday junction. For Iso I (Fig. 5a, c and e), the dye attached to the A strand is in contact with high probability with DNA bases on the C strand. Similarly, for Iso II (Fig. 5b, d and f), the dye on the B strand is in contact with high probability with DNA on the D strand. Comparing Fig. 5a and b, one notable difference is that for Iso I, the dye on the B strand has a non-zero probability of being in contact with the dye on the A strand, as well as the A bases in the center of the junction. Similarly, the dye on the A strand has non-zero probabilities of being in contact with the B strand dye and bases. Conversely, there are little-to-no contacts between the A strand dye and B bases as well as between the B strand dye and A bases for Iso II. As can be seen in the snapshots in Fig. S5 and S6,† in some cases, the dyes appear to intercalate into the base regions or major grooves of the duplexes constituting the Holliday junction, indicating that some steric effects may play a role in the dye–dye interactions. The lack of short *R*_m,n_ (less than 1.5 nm) in Fig. 5 and contacts between dyes and surrounding DNA bases for Iso II in Fig. 5 may indicate a stronger propensity of dyes in the Iso I configuration to overcome the steric effects of the DNA to form J-dimers. Furthermore, contacts between the DNA bases on the 3′ end of the dyes (*i.e.*, bases 14 and 15) for the Iso I configurations are consistent with dimer formation and low *R*_m,n_. In other words, contacts are observed between bases 14 and 15 on the A and B strands for Iso I, which are missing in Iso II. Overall, the evidence for DNA base hindrance on dye aggregation may explain the lack of smaller *R*_m,n_, despite the dyes being attached near the center of the Holliday junction.

### 3.2 Transverse dimers

**Fig. 6:**
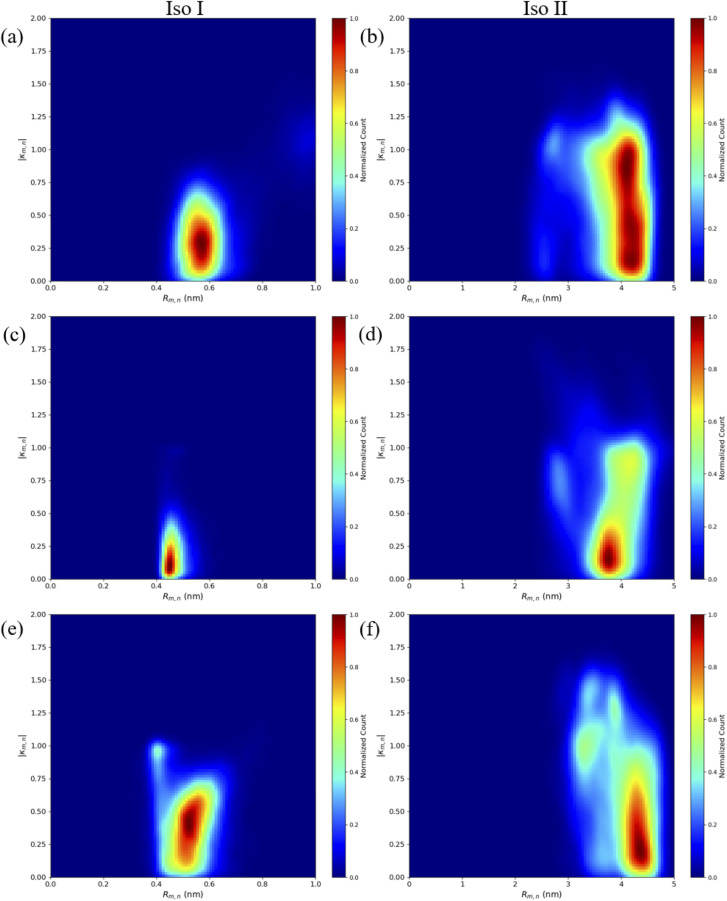
Heatmap plots of the |*κ*_m,n_| calculated using eqn (1)*versus R*_m,n_ for (a) A_Cy5_C_Cy5_ Iso I, (b) A_Cy5_C_Cy5_ Iso II, (c) A_Cy5.5_C_Cy5.5_ Iso I, (d) A_Cy5.5_C_Cy5.5_ Iso II, (e) A_Cy5_C_Cy5.5_ Iso I, and (f) A_Cy5_C_Cy5.5_ Iso II.

Similar to the adjacent dimer systems, heatmap plots of |*κ*_m,n_| *versus R*_m,n_ for the transverse dimers are presented in Fig. 6 for both simulation trials 1, 2, and 3. Transverse dimer orientations plotted as a function of simulation time for trials 1, 2, and 3 are given in Fig. S7.† Like the adjacent dimers, the Iso I and Iso II transverse dimers show distinct differences compared to one another. For the A_Cy5_C_cy5_ Iso I system shown in Fig. 6a, there is a single, prominent peak at around *R*_m,n_ ≈ 0.57 nm and |*κ*_m,n_| ≈ 0.4, indicating a closely spaced, H-like dimer configuration. Conversely, the A_Cy5_C_cy5_ Iso II dimer exhibits minimal evidence of dye–dye interactions with the prominent peak in Fig. 6b at around *R*_m,n_ ≈ 4.2 nm and |*κ*_m,n_| ≈ 0.2–1.1. As shown in Fig. 1, this is expected since the A and C strands are closely spaced for Iso I Holliday junctions and the furthest away for Iso II. The same trend is observed for A_Cy5.5_C_Cy5.5_ and A_Cy5_C_Cy5.5_ Iso I and Iso II dimers. The major peaks in the Iso I configuration occur at *R*_m,n_ ≈ 0.43 nm and |*κ*_m,n_| ≈ 0.2 for A_Cy5.5_C_Cy5.5_ and *R*_m,n_ ≈ 0.55 nm and |*κ*_m,n_| ≈ 0.50 for A_Cy5_C_Cy5.5_. Notably, the spread of *R*_m,n_ and |*κ*_m,n_| values is the tightest for A_Cy5.5_C_Cy5.5_. Like the A_Cy5_C_Cy5_ Iso II case, the major peaks in the Iso II configuration occur at *R*_m,n_ ≈ 3.8 nm and |*κ*_m,n_| ≈ 0.2–1 for A_Cy5.5_C_Cy5.5_ and *R*_m,n_ ≈ 4.4 nm and |*κ*_m,n_| ≈ 0.2–1 for A_Cy5_C_Cy5.5_. Snapshots corresponding to the heatmaps in Fig. 6 are shown in Fig. S10 and S11.† As seen in Fig. S10,† the transverse dimers are predominantly outside of the DNA base region in the center of the Holliday junction. This behavior is different from the adjacent dimers that show an evidence of intercalation and DNA base hindrance on dye aggregation. Conversely, as seen in Fig. S11,† the dyes are located on the opposite sides of the DNA Holliday junction without any apparent dye–dye interactions.

**Fig. 7:**
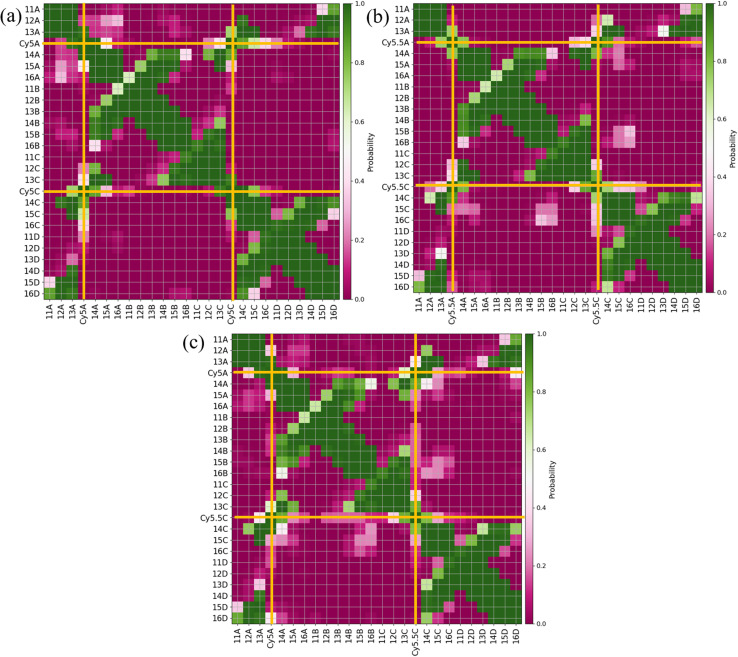
Contact maps for (a) A_Cy5_C_Cy5_ Iso I, (b) A_Cy5.5_C_Cy5.5_ Iso I, and (c) A_Cy5_C_Cy5.5_ Iso I. Two residues are considered to be in contact if they are within 1.2 nm. The *x*- and *y*-axes correspond to the dye or base residue names. Dye contacts with all other residues are indicated in the plots with bold orange lines.

Similar to what was done for the adjacent dimers, contact maps were generated for the transverse dimers in the Iso I Holliday junction configuration, shown in Fig. 7. The Iso I configuration is presented since the Iso II does not show any evidence of dye–dye interactions (*i.e.*, Iso II exhibits large *R*_m,n_). The contact maps for the Iso II configurations are shown in Fig. S12.† The most notable difference compared to the adjacent dimers are the much higher probabilities for the dyes to be in contact for the transverse, Iso I dimer systems (as seen from the intersections of the A and C dyes in Fig. 7). Another notable difference is the lack of dye contacts with DNA strands that a dye is not attached to. For transverse Iso I systems, the dyes are predominantly in contact with DNA bases on the same strand the dyes are attached to, as well as with the dye and DNA bases on the transverse strand.

### 3.3 Comparison with experiment

To compare computational results with available experimental data, *J*_m,n_ values were calculated for the transverse, Iso I dimers, since those dimers showed the smallest *R*_m,n_ and largest amounts of dye–dye interactions (*i.e.*, strong excitonic coupling). Currently, dimer experimental data for |*J*_m,n_| and *R*_m,n_ only exists for transversely attached dyes. The |*J*_m,n_| values were calculated using eqn (2) and the transition dipole moments (*μ*_i_) for Cy5 and Cy5.5 were calculated using TD-DFT, with values of *μ*_Cy5_ = 15.35 D and *μ*_Cy5.5_ = 15.57 D.^51,56^ The values of *r* and *s* in eqn (2) were chosen to be the ends of the dye along the long axis, resulting in dye lengths of about 1.4 nm and 1.5 nm for Cy5 and Cy5.5, respectively.

**Fig. 8:**
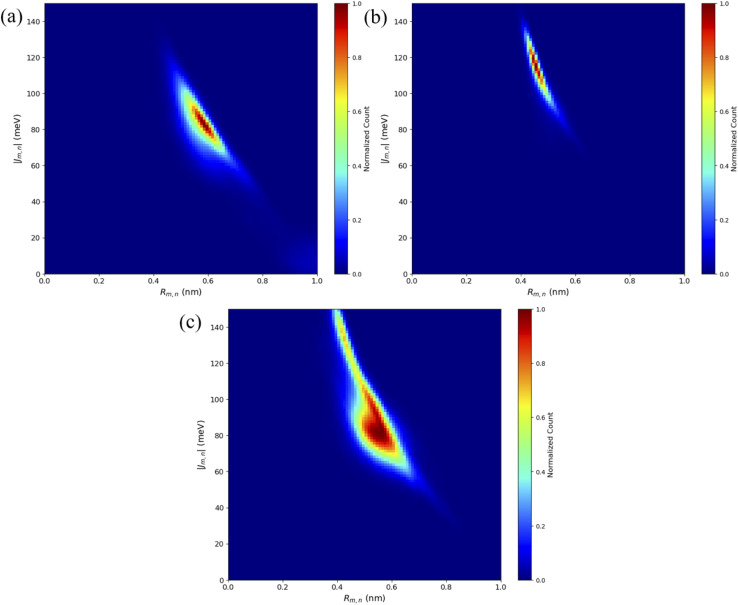
Heatmap plots of the |*J*_m,n_| calculated using eqn (2)*versus R*_m,n_ for (a) A_Cy5_C_Cy5_ Iso I, (b) A_Cy5.5_C_Cy5.5_ Iso I, and (c) A_Cy5_C_Cy5.5_ Iso I.

**Fig. 9:**
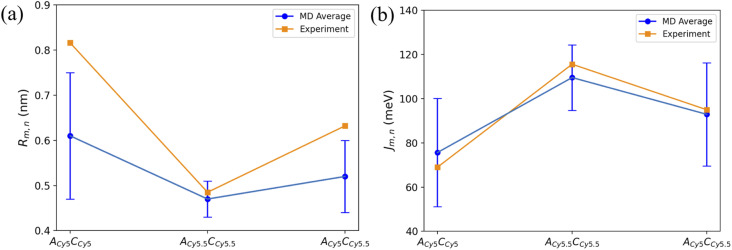
Plots of (a) *R*_m,n_ and (b) |*J*_m,n_| obtained from MD simulations and experiment. MD simulation results were obtained by averaging the data over the 900 ns simulations. Error bars denote the standard deviation of the average MD values. Experimental data acquired from Chowdhury *et al.*^47^ Lines connecting the points are to denote trends in the data, not fits.

As expected, based on the *R*_m,n_ values in Fig. 6, the |*J*_m,n_| values increase in the order of A_Cy5_C_Cy5_, A_Cy5_C_Cy5.5_, and A_Cy5.5_C_Cy5.5_. The relative magnitudes and orientations of the transition dipole moments also play a role in the trend of |*J*_m,n_| values since *μ*_Cy5.5_ is slightly larger than *μ*_Cy5_, however, that is somewhat mitigated by the longer dye length for Cy5.5. Furthermore, as seen in Fig. 8, the A_Cy5.5_C_Cy5.5_ system has the smallest spread of *R*_m,n_ and |*J*_m,n_| values compared to A_Cy5_C_Cy5_ and A_Cy5_C_Cy5.5_. That being said, A_Cy5_C_Cy5.5_ exhibits |*J*_m,n_| comparable to A_Cy5.5_C_Cy5.5_, indicating instances of more closely spaced and stronger coupled dimer configurations. Fig. 9 shows the average *R*_m,n_ and |*J*_m,n_| obtained from the three trials of the A_Cy5_C_Cy5_, A_Cy5.5_C_Cy5.5_, and A_Cy5_C_Cy5.5_ in the Iso I configurations compared to the experimentally available values obtained using the KRM model.^47^ The average MD results, standard deviations, and experimental values are given in Table 1. Overall, the average values obtained from the MD simulations follow the experimental trends, with A_Cy5_C_Cy5_ having the largest *R*_m,n_ and smallest |*J*_m,n_|, and A_Cy5.5_C_Cy5.5_ having the smallest *R*_m,n_ and largest *J*_m,n_. Additionally, all of the experimental |*J*_m,n_| values fall within the MD average standard deviations. The MD simulations underestimate *R*_m,n_ values for the transverse, Iso I systems, however the simulations could reproduce the experimental trends. This may be due to differences in how the KRM model treats the transition dipole moment vectors compared to how we defined them based on the molecular structure of the dye – slight bending or stretching of the dyes during the MD simulations may cause small fluctuations in the transition dipole moments and the resultant *R*_m,n_. Furthermore, Fig. 9 shows that the standard deviations for A_Cy5.5_C_Cy5.5_ are smaller than A_Cy5_C_Cy5_ and A_Cy5_C_Cy5.5_. It suggests that the transverse Cy5.5 dimer are stabilized to be a more closely pack and strongly coupled dimer, which may be due to the stronger dye–dye interactions for the Cy5.5 homodimer.

**Table 1:** Average MD results for the three simulation trials over the entire 900 ns trajectories along with their standard deviations. Experimental results are given in brackets and were obtained from Chowdhury *et al.* who used the KRM model to extract *R*_m,n_ and |*J*_m,n_| from experimental data.^47^

System	*R*_m,n_ (nm)	|*J*_m,n_| (meV)
A_Cy5_C_Cy5_ Iso I	0.61 ± 0.14 [0.816]	75.64 ± 24.50 [69.0]
A_Cy5.5_C_Cy5.5_ Iso I	0.47 ± 0.04 [0.485]	109.56 ± 14.81 [115.6]
A_Cy5_C_Cy5.5_ Iso I	0.52 ± 0.08 [0.632]	92.92 ± 23.35 [95.0]

## 4. Discussion

MD simulations of the Cy5 and Cy5.5 homodimers and heterodimers attached to adjacent Holliday junction DNA strands show that the Holliday junction isomer plays a major role in the resultant dye orientations. For all systems in this study, there are a variety of dye orientations that occur, indicating some structural heterogeneity that differs between Iso I and Iso II configurations. Furthermore, as seen in Fig. S3, S4, S8, and S9,† the Holliday junction inter-duplex angles (IDAs) and twist angles (angles between the constituent duplexes), calculated according to Fig. S1,† show a wide range of values. Typical Holliday junction IDAs and twist angles fall in the range of ∼60° and ∼40–60°, respectively.^57–59,82^ It should be noted that these values are for Holliday junctions without dyes attached to the DNA backbone – the dyes in our study act as an additional base or spacer in the DNA sequence, which may affect the DNA dynamics and conformations. This could lead to discrepancies observed between pure DNA Holliday junctions and Holliday junctions with dyes. The IDA and twist angle values still indicate the Holliday junctions for most systems predominantly remain in the stacked Iso I or Iso II configurations.

Based on the diagram in Fig. 1, the initial inter-dye separations and conditions of the adjacent dimers should be approximately the same for both Iso I and Iso II configurations. However, as shown in Fig. 3, the Iso I Holliday junction configurations preferentially form J-like dimers while the Iso II configurations do not. For adjacent dimers on the Iso II Holliday junction, the values of *R*_m,n_ are in the range of 1.5–2.8 nm. Furthermore, they are oriented predominantly in an H-like fashion with some obliqueness, as indicated by the |*κ*_m,n_| values ranging from 0–1, but do not form closely spaced dimers. Conversely, the adjacent dimers attached to the Iso I Holliday junctions achieve a smaller *R*_m,n_ of approximately 1.4 nm. The main peak in the heatmaps of Fig. 3b, d and e correspond to J-like stacking of the dyes as indicated by the range of |*κ*_m,n_| from 1.25–1.75. Compared to the adjacent dimer systems, the transverse dimer systems either form closely spaced H-like aggregates (Iso I) or don't form dimers at all (Iso II), which is intuitive based on Fig. 1. However, as can be seen in Fig. 8 and 9, A_Cy5.5_C_Cy5.5_ has the smallest spread of *R*_m,n_ and |*J*_m,n_|, which may be due to the stronger dye–dye interactions between the Cy5.5 dyes that “lock” them into the closely spaced, H-like configuration. This is also highlighted by the smaller standard deviations for the average *R*_m,n_ and |*J*_m,n_| values of A_Cy5.5_C_Cy5.5_ compared to A_Cy5_C_Cy5_ and A_Cy5_C_Cy5.5_. This may possibly be explained by the larger conjugated Cy5.5 structure compared to Cy5, enhancing the dye–dye interactions. Furthermore, we have shown *via* DFT calculations of the partition coefficient in water and *n*-octanol that Cy5.5 is slightly more hydrophobic than Cy5, which may also lead to the smaller *R*_m,n_ and the larger |*J*_m,n_|.^56^ The differences between the adjacent dimers and transverse dimers in the Iso I configurations may also be explained in part by the contact maps shown in Fig. 5 and 9. For adjacent dimers in the Iso I configuration, there exist numerous contacts between the dyes and the DNA bases in the center of the Holliday junction, as well as the bases flanking the dyes. This indicates that to form a dimer (in this case, J-like dimers), the dyes need to overcome the barriers set by the surrounding DNA. Conversely, the transverse dimers exist in the center of the Holliday junction and do not show many contacts with the surrounding DNA, which may otherwise limit closely spaced dimer formation.

Our adjacent dimer MD data agrees qualitatively with experimental results from Cannon *et al.*^46^ that showed red-shifted absorption peaks for adjacent Cy5 homodimers in Holliday junctions and small amounts of Davydov splitting, indicating J-like dye orientations with some obliqueness. Similar results were obtained by Huff *et al.*^41^ However, the presence of dye orientations at large inter-dye distances (>2.5 nm) suggests that the dyes may not always form closely coupled dimers when they are bound to adjacent DNA strands. Experimentally, it was found that Holliday junctions with two Cy5 dyes attached to adjacent DNA arms exhibit lower denaturing temperature compared to that of unlabeled junctions, indicating weaker dye–dye interactions and larger *R*_m,n_.^41^ Furthermore, it has been shown that there exists red-shifted and blue-shifted absorption features for the adjacent dimers, suggesting that there are subpopulations of J- and H-like dimers in the solution.^41^ Similar results were found for heterodimers consisting of Cy5 and Cy5.5 dyes.^47^ Absorption results of the Cy5–Cy5.5 heterodimer suggest structural heterogeneity in the dye–DNA solutions with subpopulations of excitonically and non-excitonically interacting dyes.^47^ Our MD results suggest that, depending on if the Holliday junction is in the Iso I or Iso II configuration, adjacent dimers can adopt both H-like and J-like arrangements with *R*_m,n_ larger than 1.4 nm. The DNA surrounding the dyes near the center of the junction may inhibit the dyes achieving smaller *R*_m,n_ values, as shown in the contact map plots. These results coincide with experimental findings of structural heterogeneity and larger inter-dye separations.^41,46,47^

Compared to the adjacent dimers, the transverse dimers exhibit two vastly different dye orientations depending on the Holliday junction configuration. Transverse dimers attached to the Iso II Holliday junctions exhibit *R*_m,n_ predominantly over 4.0 nm, suggesting weak dye–dye interactions. Experimental measurements of transverse Cy5–Cy5.5 heterodimers suggest that there may exist subpopulations of monomers in the solution. However, experimental evidence suggests that Iso I dominates.^41,47^ Since the experimental measurements include the ensemble average of the system, the subpopulation of weakly interacting dyes may still be present in the experimental spectra and resultant KRM data. This could explain the larger *R*_m,n_ values observed experimentally compared to the MD results since only Iso I conformers were considered for the results presented in Fig. 8 and 9. If a small percentage of Iso II data was included in the *R*_m,n_ and *J*_m,n_ calculations, better agreement with experiment might be obtained. Based on our MD results, these subpopulations may be occurring due to the Holliday junctions organizing into the Iso II configuration, which results in essentially two isolated monomers. The Iso I Holliday junctions, however, yield dye orientations that are predominantly H-like with much smaller *R*_m,n_ compared to both the adjacent dimers and Iso II transverse dimers. Furthermore, there is a lack of interactions of the dyes with the opposing DNA strands for the transverse, Iso I configurations, allowing for closer inter-dye distances. The H-like stacking and *R*_m,n_ of 0.4–0.7 nm lead to |*J*_m,n_| values of 60–150 meV. These results agree well with the experimental evidence for H-like transverse Cy5 and Cy5.5 dye dimers, showing small *R*_m,n_ values and thus large |*J*_m,n_|, consistent with our MD results as shown in Fig. 9 and Table 1.^41,46,47^ Additionally, it was found experimentally that DNA Holliday junctions with transversely attached Cy5 dyes have higher denaturing temperatures compared to adjacently attached dyes, indicating stronger dye–dye interactions.^41^ In the future, we will explore the role of DNA Holliday junction dynamics and isomerization in-depth with more advanced sampling methods.

## 5. Conclusions

MD simulations were carried out to examine the role of dye attachment locations and DNA Holliday junction isomerization on dimer orientations. Dimers consisting of either two Cy5 dyes, two Cy5.5 dyes, or one each of Cy5 and Cy5.5 dyes, were attached to either adjacent or transverse strands of DNA Holliday junctions in the stacked, Iso I or Iso II configurations. Overall, Iso I configurations agree well with experimental measurements. Adjacent dimers in the Iso I configuration exhibits predominantly J-like dye orientations while the Iso II configuration exhibits H-like dimers with large *R*_m,n_. For the dye–DNA interactions, we found that dyes intercalate and stack with surrounding DNA bases, which might hinder dimer formation. Transverse dimers in the Iso II configuration exhibit little evidence of dye–dye interactions and essentially behave like independent monomers. Conversely, transverse dimers in the Iso I configuration have the smallest *R*_m,n_ and exhibit H-like stacking with negligibly few dye–DNA interactions. Furthermore, the calculated |*J*_m,n_| for transverse, Iso I systems agree well with experiments. Our MD results reveal dye attachment schemes and DNA Holliday junction isomers on dye–DNA interactions, dye dynamics, and resultant dimer orientations, which can guide the synthesis and experimental studies for tailoring the dye–DNA structures towards applications that exploit dye aggregates and exciton dynamics.

## Funding

Molecular dynamics research was supported by the Department of the Navy, Office of Naval Research (ONR), *via* ONR award no. N00014-19-1-2615. Modeling was performed at the High Performance Computing Center at Idaho National Laboratory, which is supported by the Office of Nuclear Energy of the U.S. Department of Energy and the Nuclear Science User Facilities, under Contract No. DE-AC07-05ID14517. Further computational modeling and data analysis were performed on the computing servers sponsored by the U.S. Department of Energy (DoE), Office of Basic Energy Sciences, Division of Materials Science and Engineering through the Established Program to Stimulate Competitive Research (EPSCoR), *via* award no. DE-SC0020089. Y.-C. K and J. S. M. of the U.S. Naval Research Laboratory (NRL) were supported by NRL base funding, the NRL Institute for Nanoscience, and ONR award no. 770 N0001419WX01811.

## Conflicts of interest

There are no conflicts of interest to declare.

## Supplementary Material

RA-012-D2RA05045E-s001
