# Supplementary material for: Molecular dynamics simulations of cyanine dimers attached to DNA Holliday junctions
Source: RSC Adv. 2022 Oct 4;12(43):28063–78. doi: 10.1039/d2ra05045e (PMC9530999; doi:10.1039/d2ra05045e)
Supplement: RA-012-D2RA05045E-s001 [file RA-012-D2RA05045E-s001.pdf]

Electronic Supplementary Material (ESI) for RSC Advances.
This journal is © The Royal Society of Chemistry 2022

Inter-duplex and Twist Angles Calculations

The inter-duplex angles (IDA) were calculated by finding the center of mass of C1' atoms on each of the two Holliday junction arms as shown in Figure S1. The center of mass of the two Holliday junction arms were used due to fluctuations observed at the ends of the arms that may lead to inaccurate values. The center of mass of the C1' atoms of the four DNA bases in the center of the Holliday junctions was used for the third point in the IDA calculation. The IDA was calculated by taking the dot product of the corresponding vectors.

For twist angles, the vectors corresponding to the two duplexes were calculated by finding the difference between the centers of mass of the C1' atoms on each of the Holliday junction arms, as shown in Figure S1. Then, the twist angle was calculated by taking the dot product of the corresponding vectors.

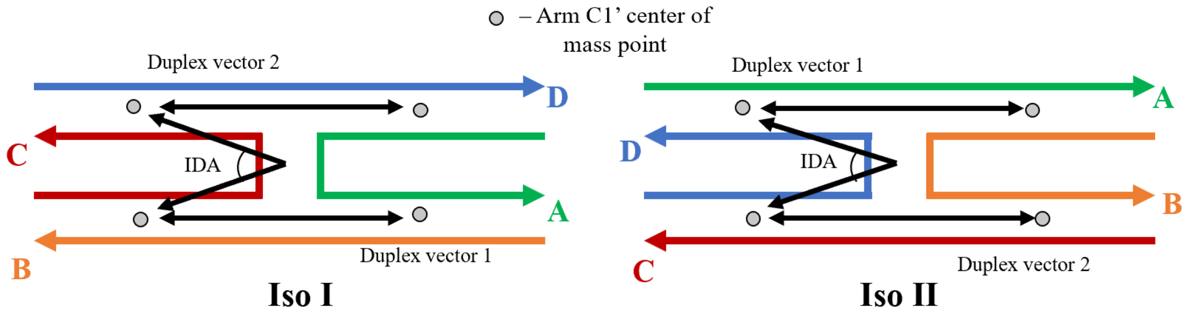

Figure S1. Diagram of the Holliday junction and corresponding inter-duplex angles (IDA) in (a) Iso I and (b) Iso II configurations. The duplex vectors used for the twist angles are shown.

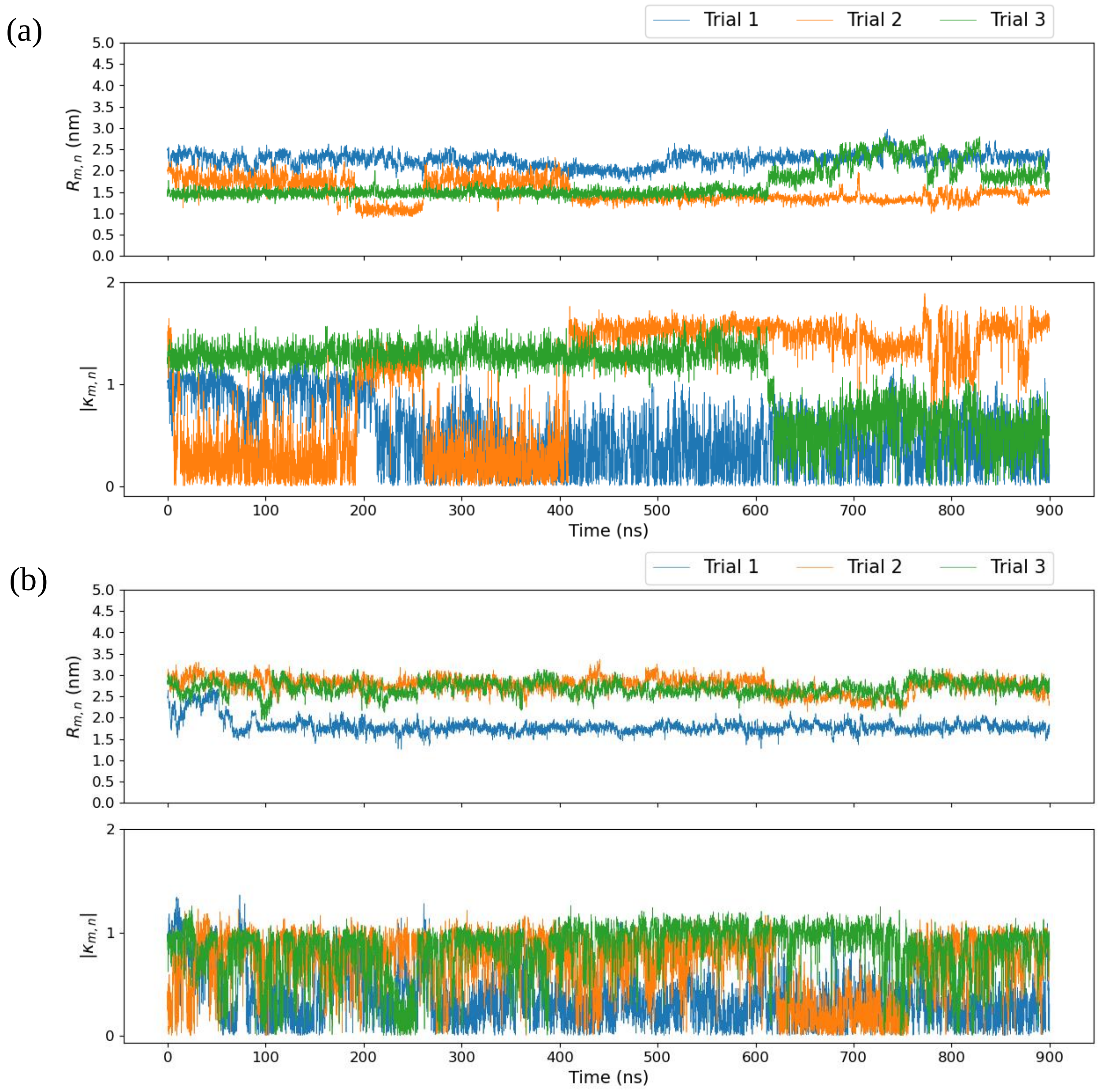
(a)
Trial 1
Trial 2
Trial 3
$R_{m,n}$ (nm)
$|\kappa_{m,n}|$
Time (ns)
(b)
Trial 1
Trial 2
Trial 3
$R_{m,n}$ (nm)
$|\kappa_{m,n}|$
Time (ns)

(c)

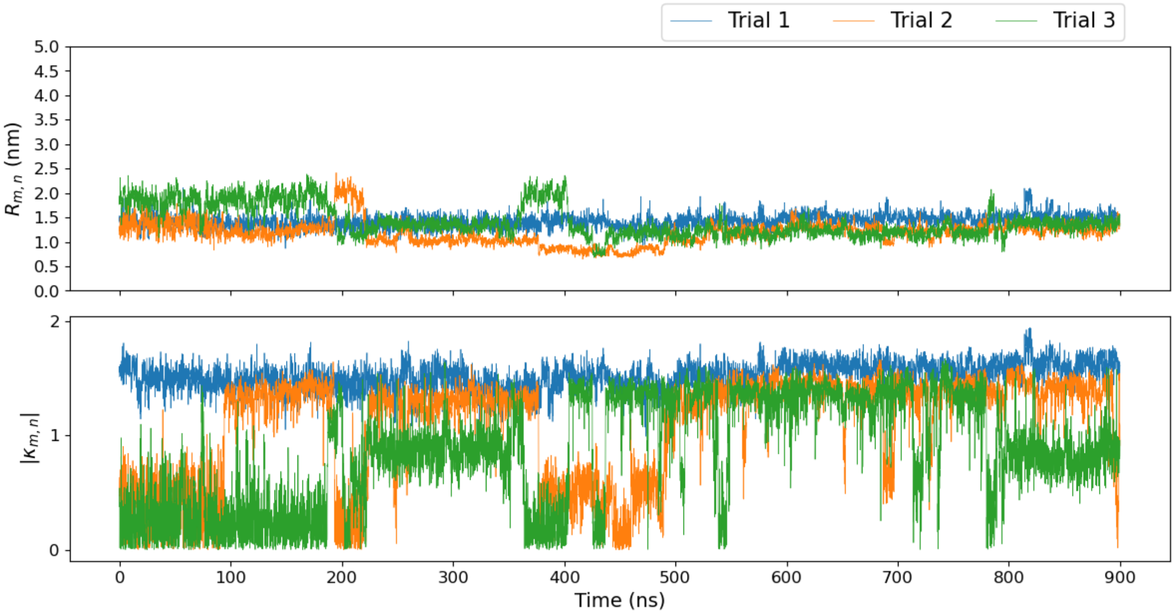

Trial 1
Trial 2
Trial 3
5.0
4.5
4.0
3.5
3.0
2.5
2.0
1.5
1.0
0.5
0.0
$R_{m,n}$ (nm)
2
1
0
$|\kappa_{m,n}|$
0 100 200 300 400 500 600 700 800 900
Time (ns)

(d)

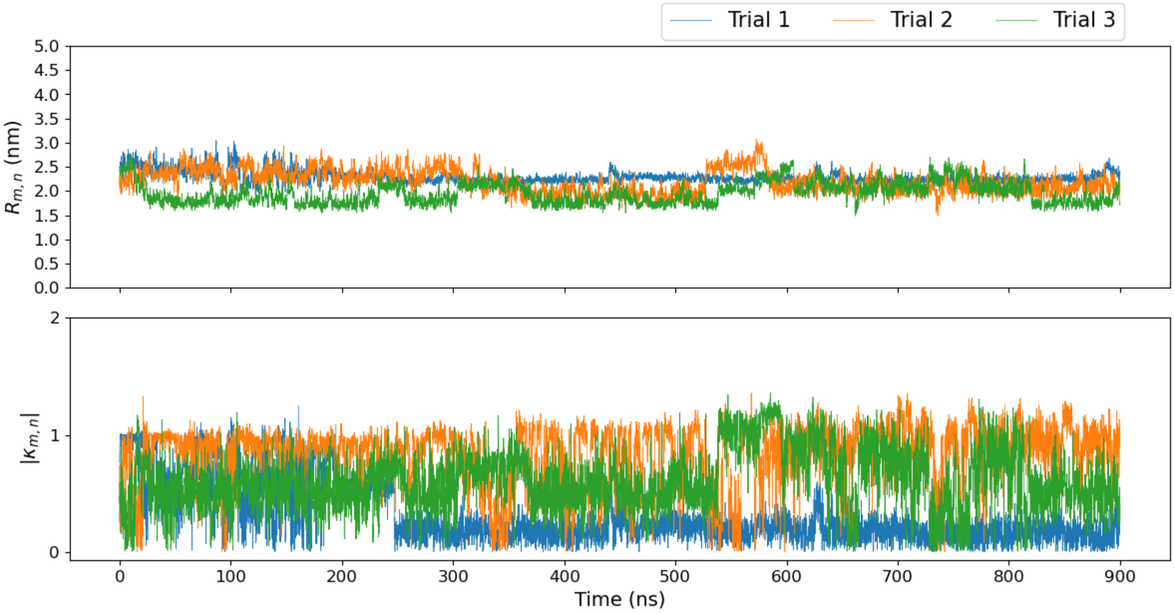

Trial 1
Trial 2
Trial 3
5.0
4.5
4.0
3.5
3.0
2.5
2.0
1.5
1.0
0.5
0.0
$R_{m,n}$ (nm)
2
1
0
$|\kappa_{m,n}|$
0 100 200 300 400 500 600 700 800 900
Time (ns)

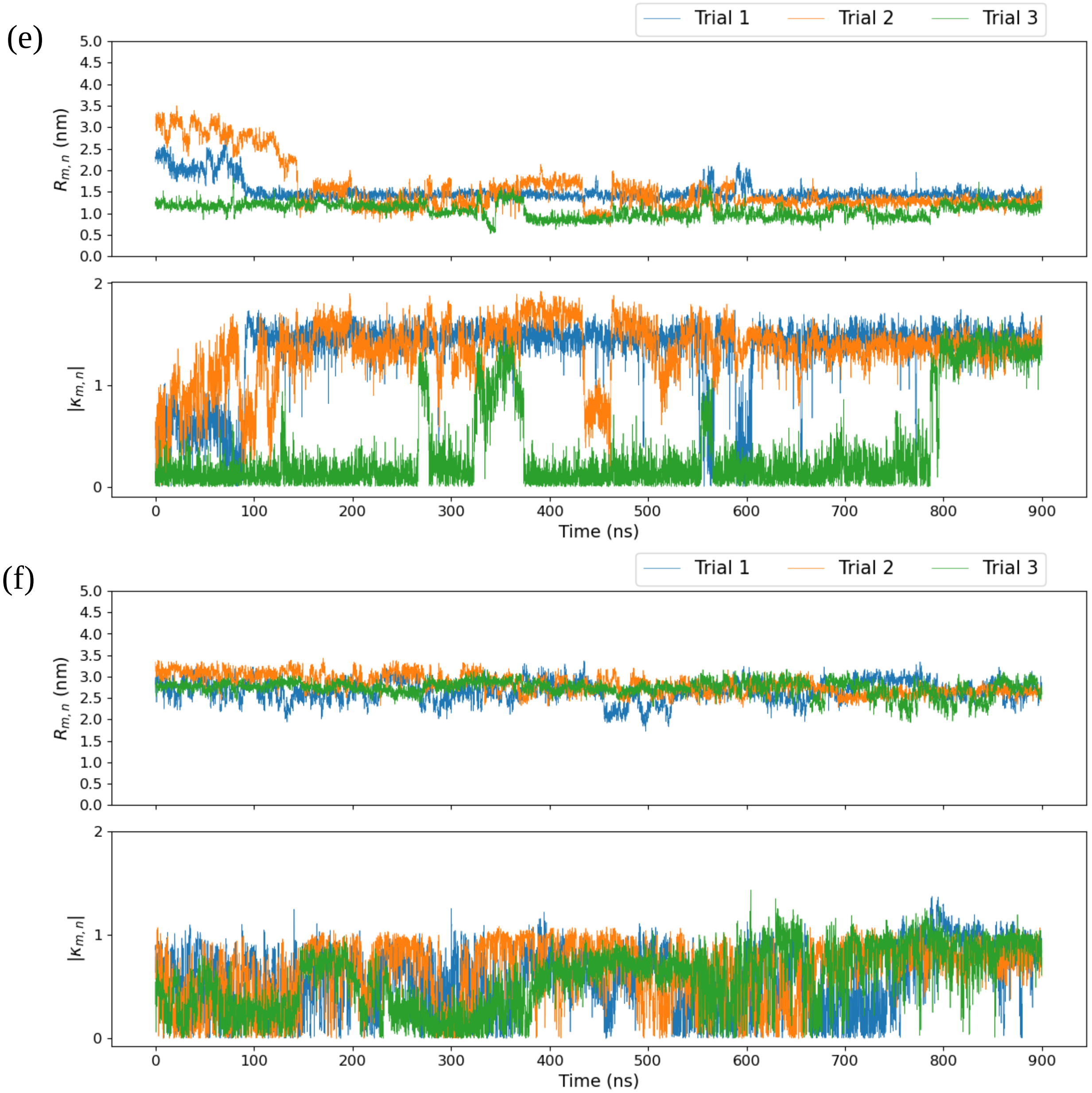

Figure S2. Dimer center-to-center distances ($R_{m,n}$) and orientation factors ($|\kappa_{m,n}|$) for MD simulation trial 1 (blue), 2 (orange), and 3 (green) as a function of simulation time for (a) $A_{Cy5}B_{Cy5}$ Iso I, (b) $A_{Cy5}B_{Cy5}$ Iso II, (c) $A_{Cy5.5}B_{Cy5.5}$ Iso I, (d) $A_{Cy5.5}B_{Cy5.5}$ Iso II, (e) $A_{Cy5}B_{Cy5.5}$ Iso I, and (f) $A_{Cy5}B_{Cy5.5}$ Iso II. Each trial consisted of a separate, 1 μs production MD simulation with different starting velocities, and the first 100 ns were taken to be an equilibration period and so were neglected.

(a)

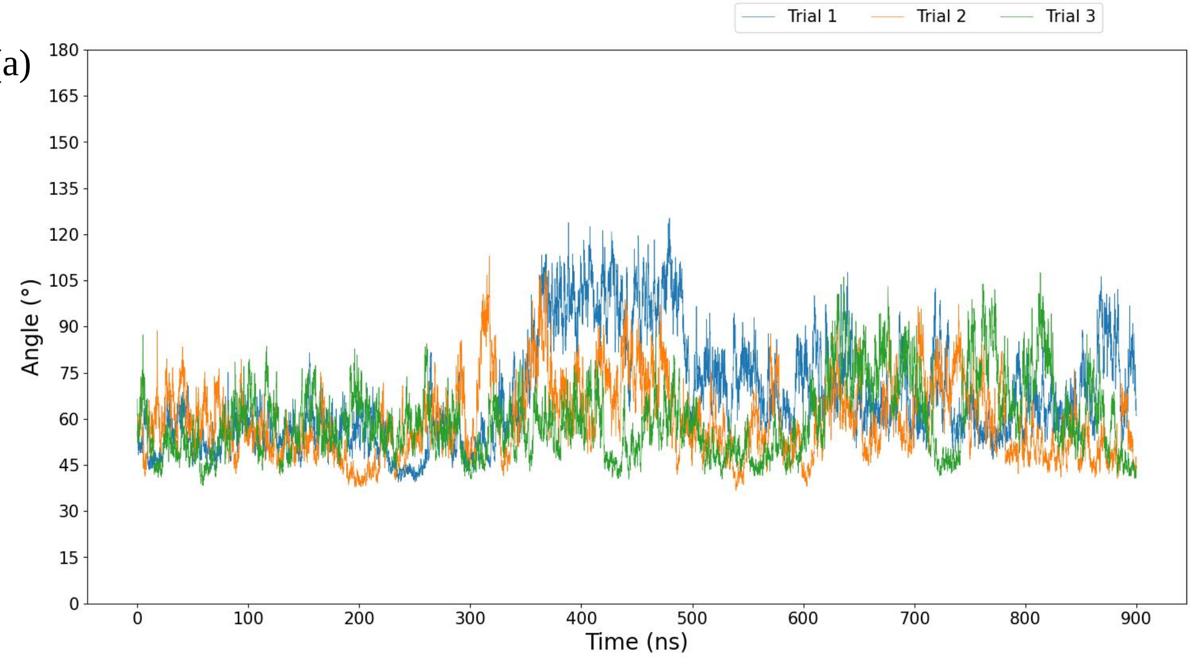
Trial 1
Trial 2
Trial 3
Angle (°)
Time (ns)
0
15
30
45
60
75
90
105
120
135
150
165
180
0
100
200
300
400
500
600
700
800
900

(b)

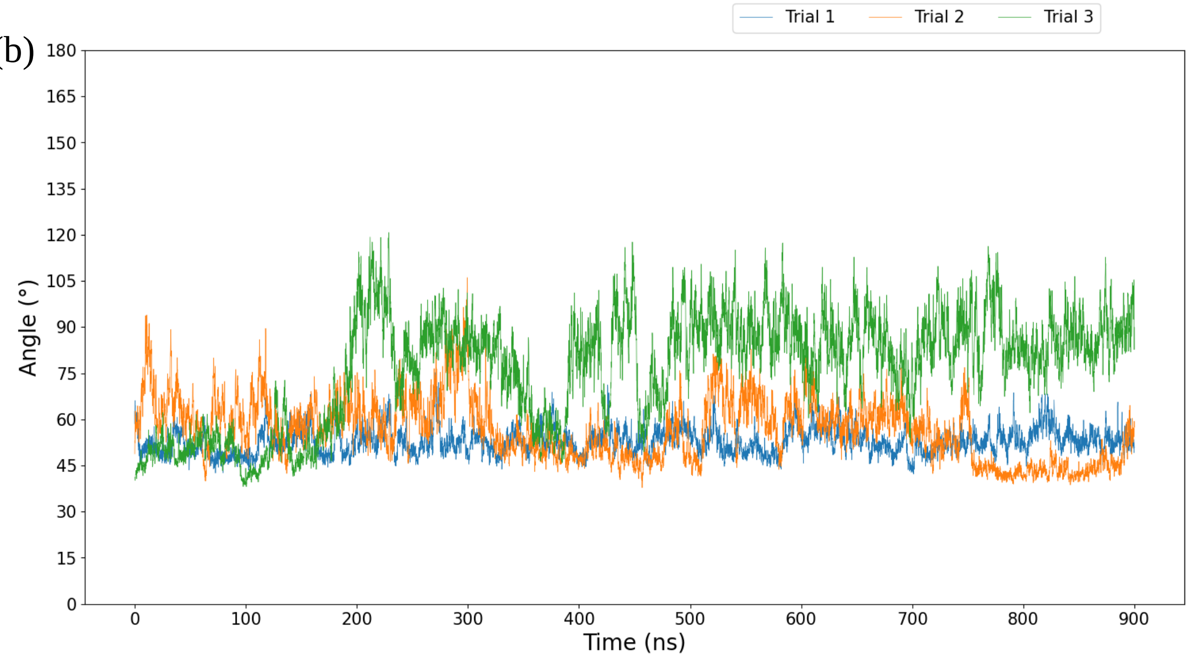
Trial 1
Trial 2
Trial 3
Angle (°)
Time (ns)
0
15
30
45
60
75
90
105
120
135
150
165
180
0
100
200
300
400
500
600
700
800
900

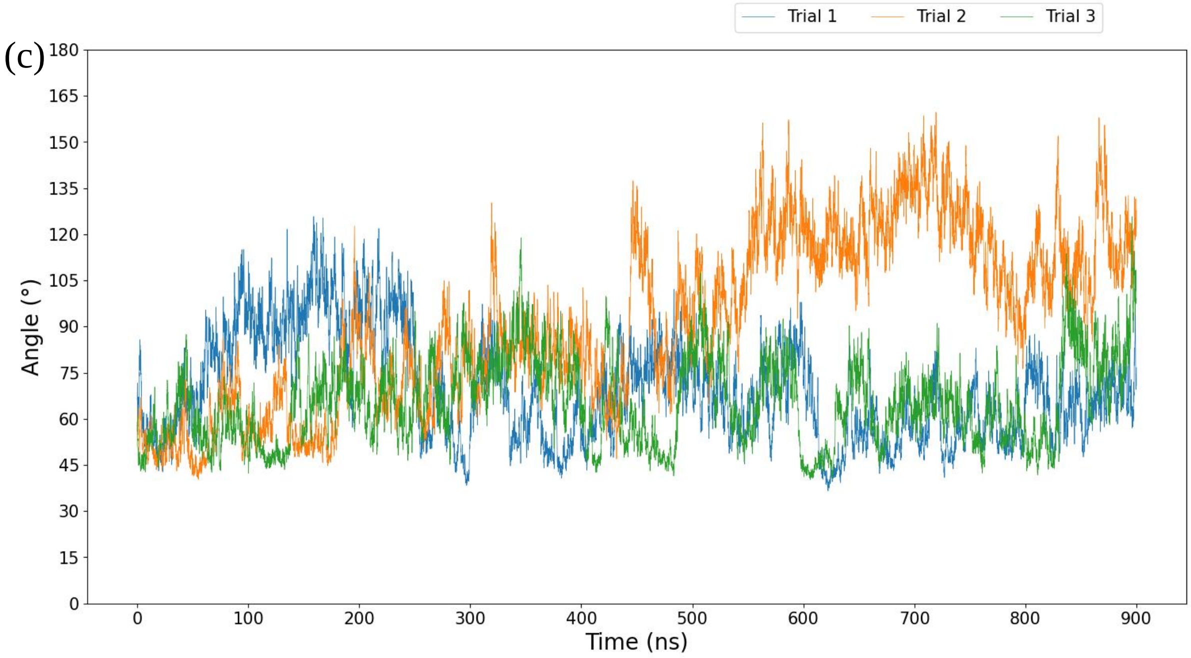
Trial 1
Trial 2
Trial 3
(c)
Angle (°)
Time (ns)
0
15
30
45
60
75
90
105
120
135
150
165
180
0
100
200
300
400
500
600
700
800
900

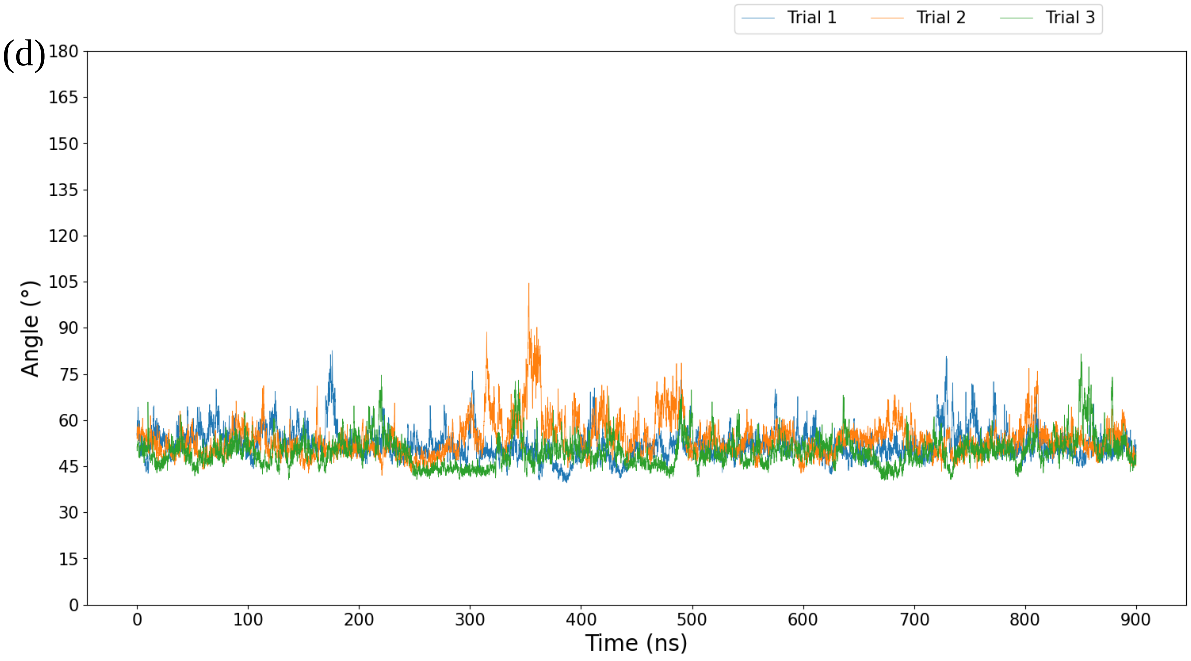
Trial 1
Trial 2
Trial 3
(d)
Angle (°)
Time (ns)
0
15
30
45
60
75
90
105
120
135
150
165
180
0
100
200
300
400
500
600
700
800
900

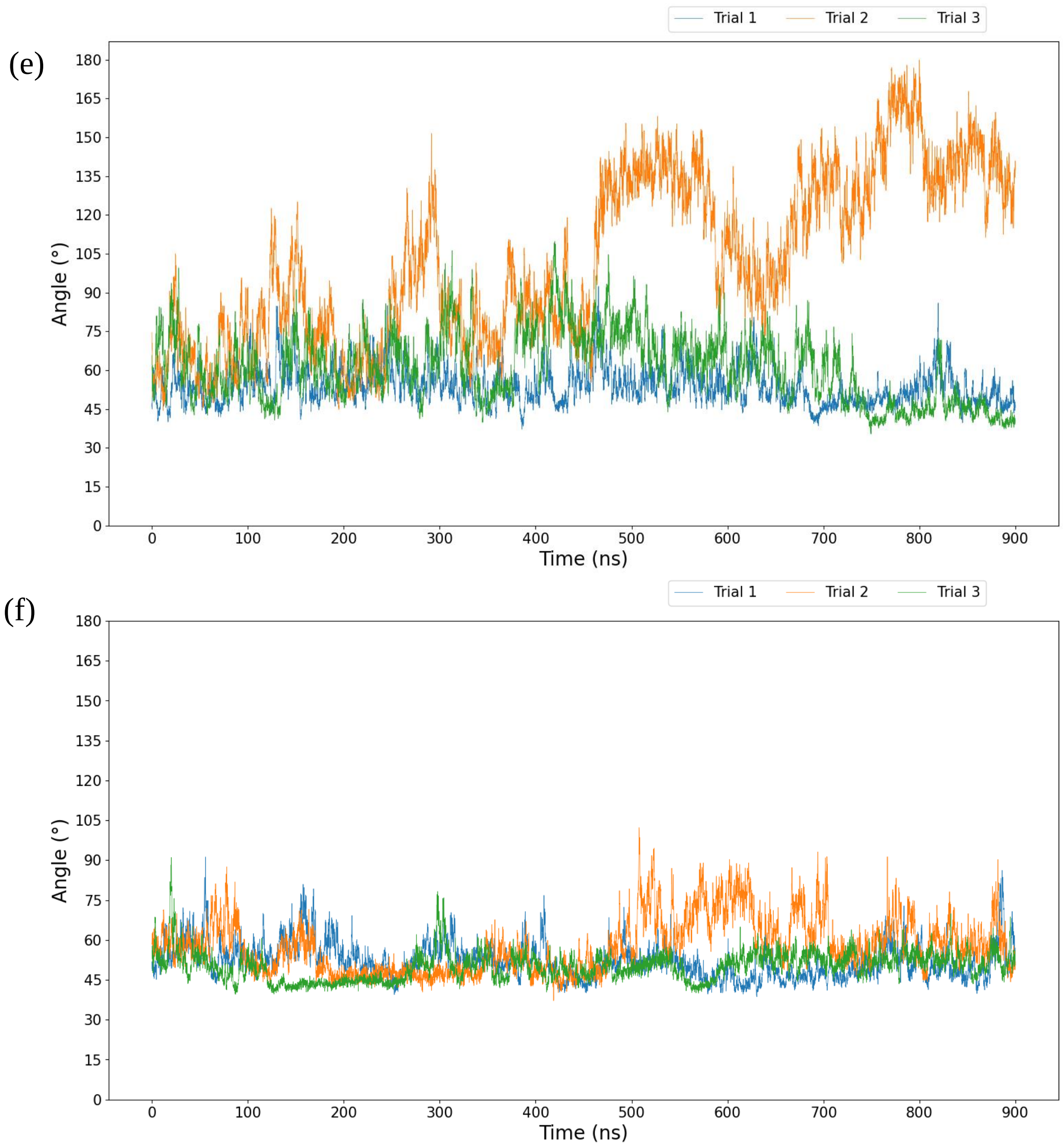

Figure S3. IDA for MD simulation trial 1 (blue), 2 (orange), and trial 3 (green) as a function of simulation time for (a) $A_{Cy5}B_{Cy5}$ Iso I, (b) $A_{Cy5}B_{Cy5}$ Iso II, (c) $A_{Cy5.5}B_{Cy5.5}$ Iso I, (d) $A_{Cy5.5}B_{Cy5.5}$ Iso II, (e) $A_{Cy5}B_{Cy5.5}$ Iso I, and (f) $A_{Cy5}B_{Cy5.5}$ Iso II. Each trial consisted of a separate, 1 μs production MD simulation with different starting velocities, and the first 100 ns were taken to be an equilibration period and so were neglected.

(a)

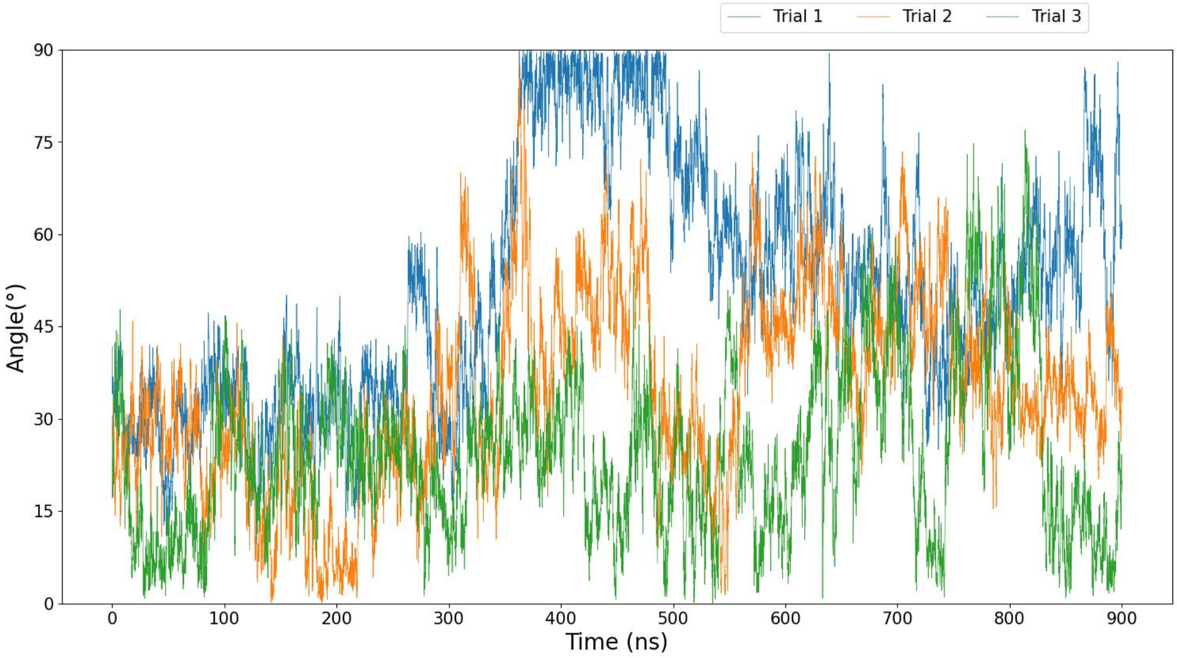
Trial 1
Trial 2
Trial 3
Angle(°)
Time (ns)
0
15
30
45
60
75
90
0
100
200
300
400
500
600
700
800
900

(b)

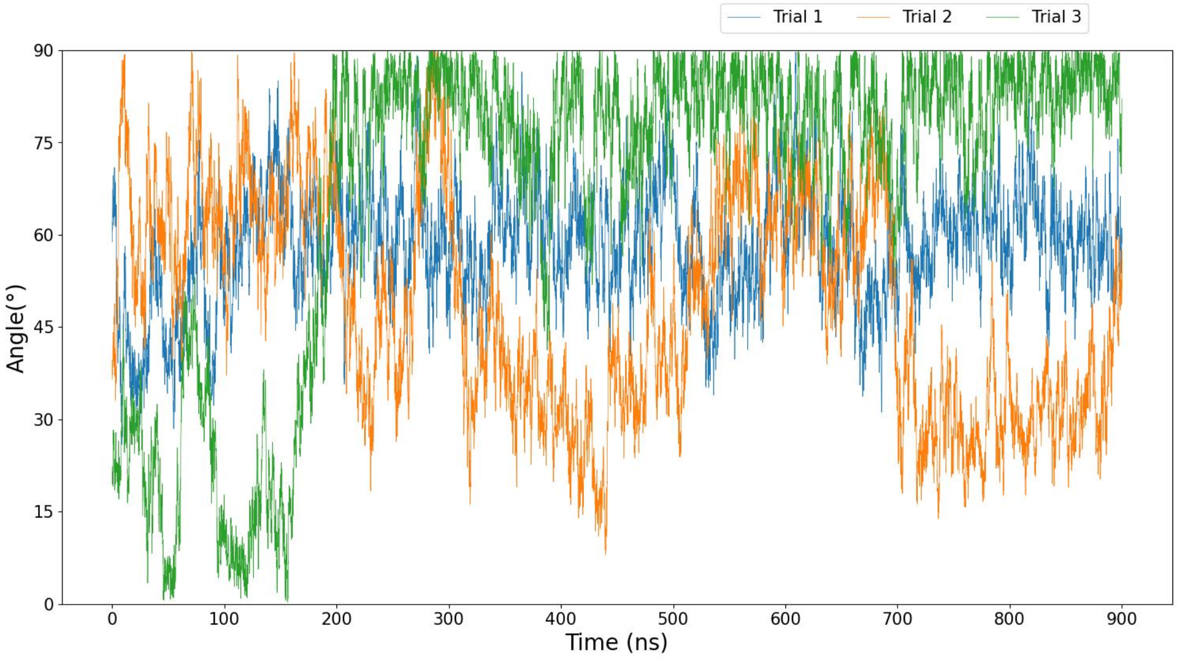
Trial 1
Trial 2
Trial 3
Angle(°)
Time (ns)
0
15
30
45
60
75
90
0
100
200
300
400
500
600
700
800
900

(c)

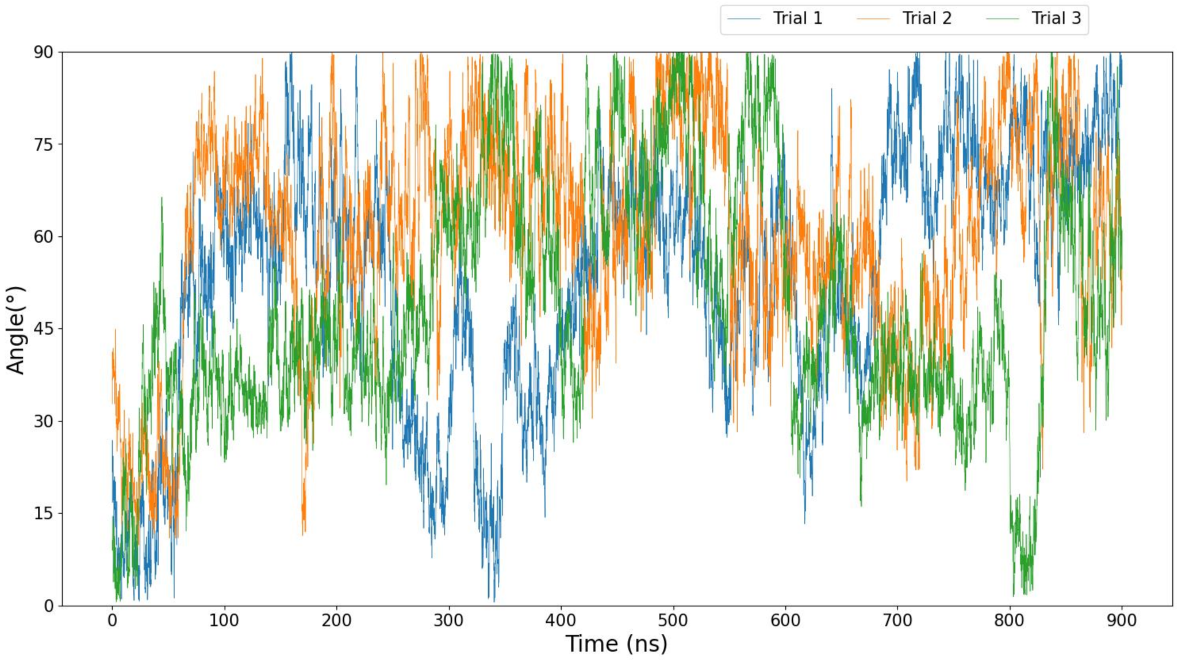

Trial 1
Trial 2
Trial 3
90
75
60
45
30
15
0
Angle(°)
0
100
200
300
400
500
600
700
800
900
Time (ns)

(d)

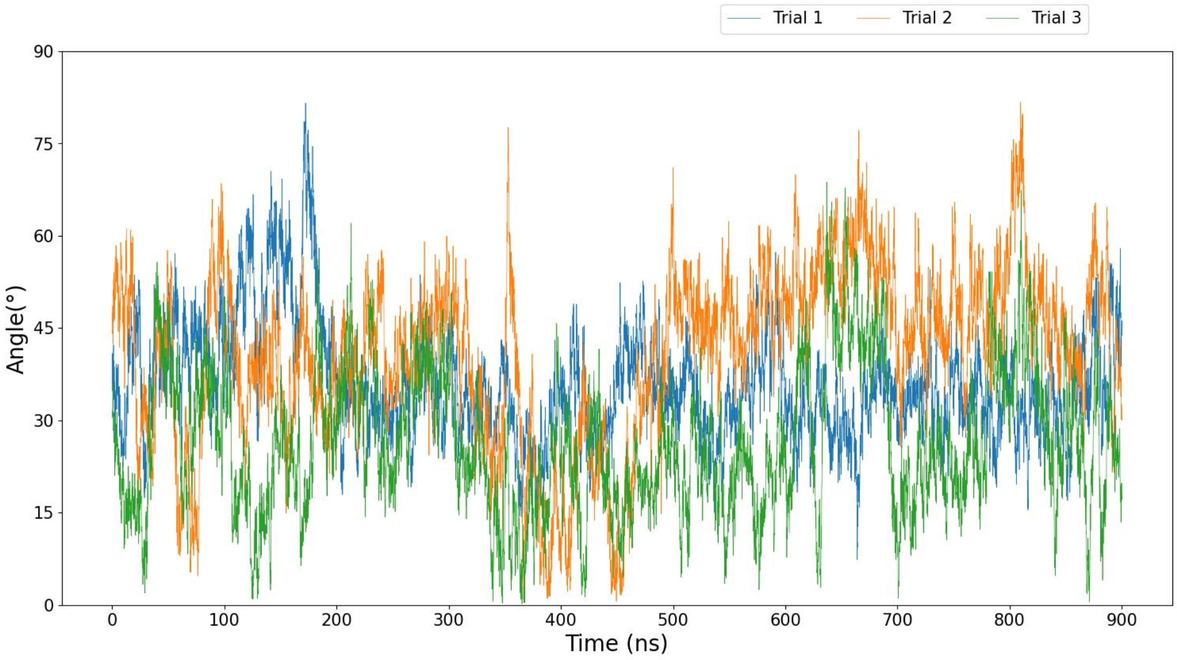

Trial 1
Trial 2
Trial 3
90
75
60
45
30
15
0
Angle(°)
0
100
200
300
400
500
600
700
800
900
Time (ns)

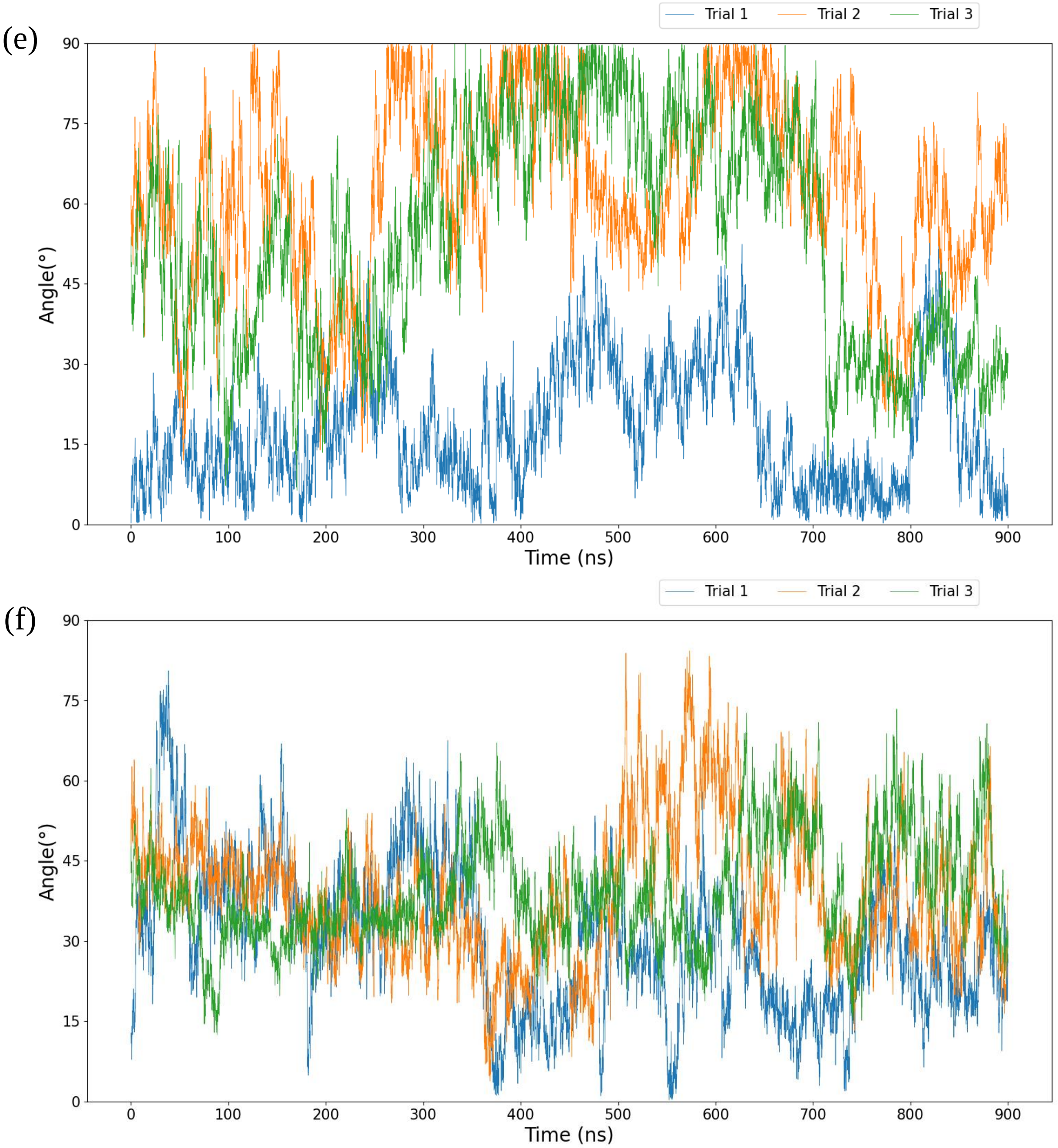

Figure S4. Holliday junction twist angles for MD simulation trial 1 (blue), trial 2 (orange), and trial 3 (green) as a function of simulation time for (a) $A_{Cy5}B_{Cy5}$ Iso I, (b) $A_{Cy5}B_{Cy5}$ Iso II, (c) $A_{Cy5.5}B_{Cy5.5}$ Iso I, (d) $A_{Cy5.5}B_{Cy5.5}$ Iso II, (e) $A_{Cy5}B_{Cy5.5}$ Iso I, and (f) $A_{Cy5}B_{Cy5.5}$ Iso II. Each trial consisted of a separate, 1 μs production MD simulation with different starting velocities, and the first 100 ns were taken to be an equilibration period and so were neglected.

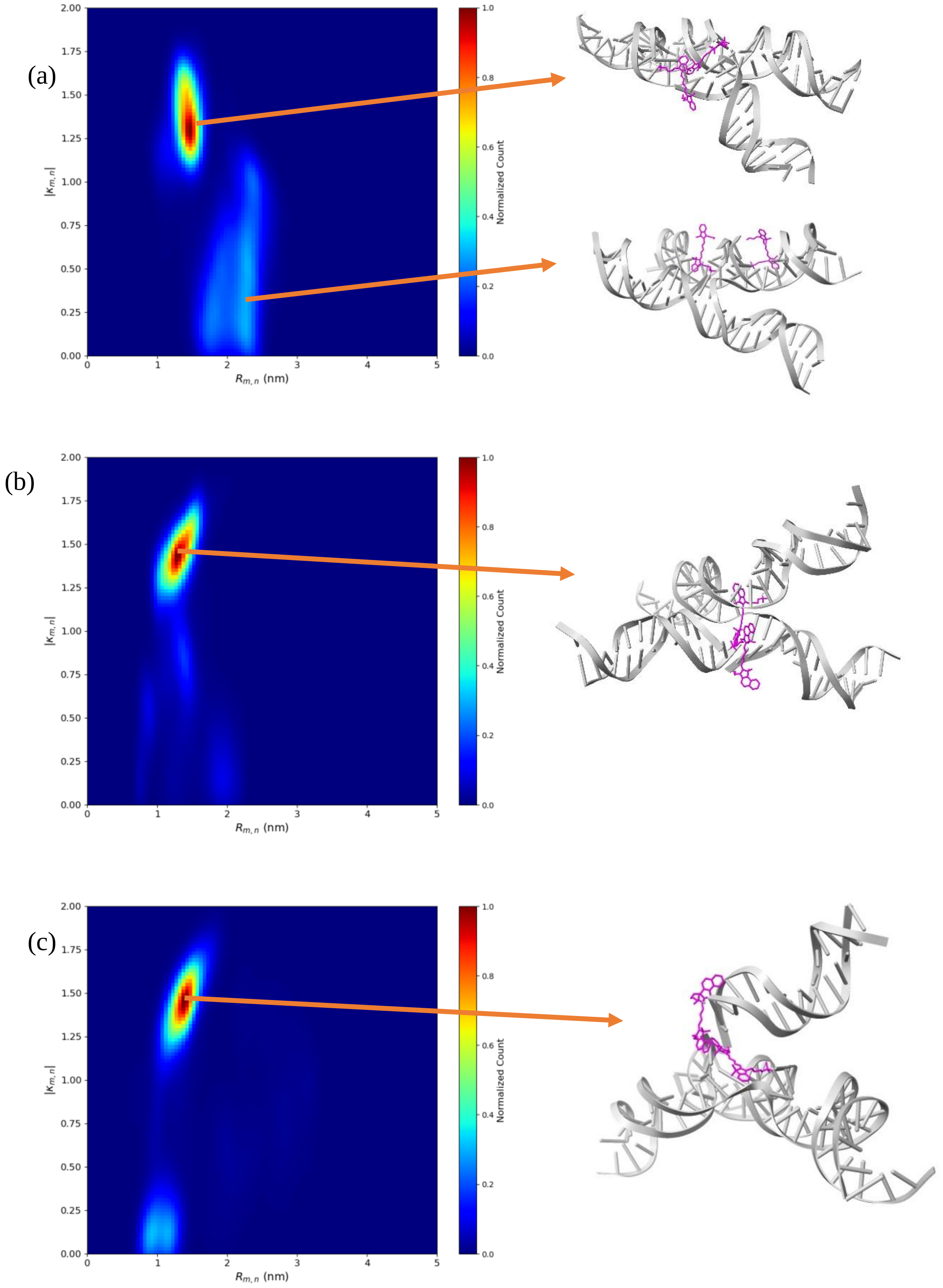

Figure S5. Heatmaps of $|\kappa|$ versus $R_{m,n}$ and corresponding representative snapshots for (a) $A_{Cy5}B_{Cy5}$ Iso I, (b) $A_{Cy5.5}B_{Cy5.5}$ Iso I, and (c) $A_{Cy5}B_{Cy5.5}$ Iso I. The dyes are colored magenta while the DNA is colored gray.

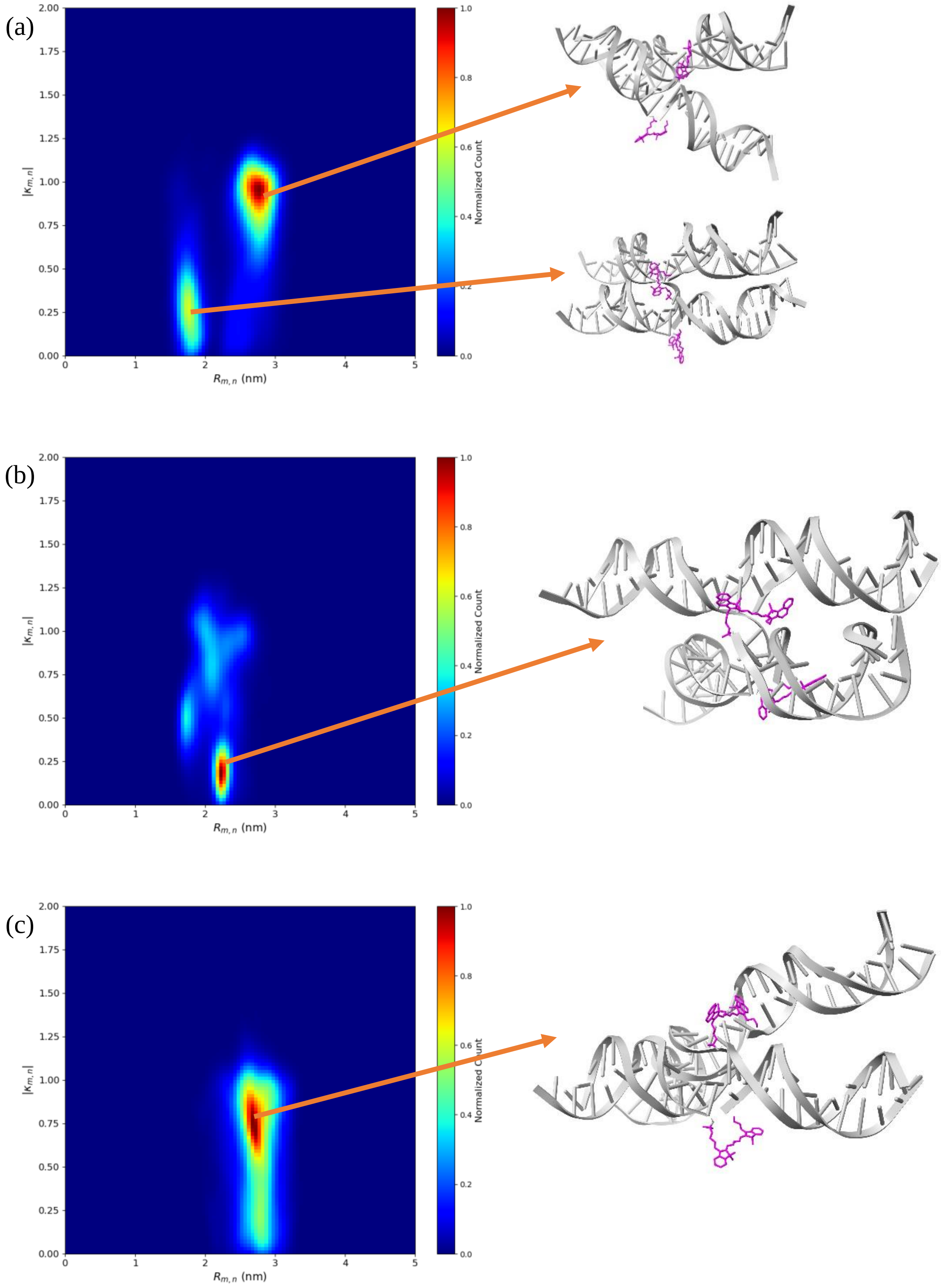

Figure S6. Heatmaps of $|\kappa|$ versus $R_{m,n}$ and corresponding representative snapshots for (a) $A_{Cy5}B_{Cy5}$ Iso II, (b) $A_{Cy5.5}B_{Cy5.5}$ Iso II, and (c) $A_{Cy5}B_{Cy5.5}$ Iso II. The dyes are colored magenta while the DNA is colored gray.

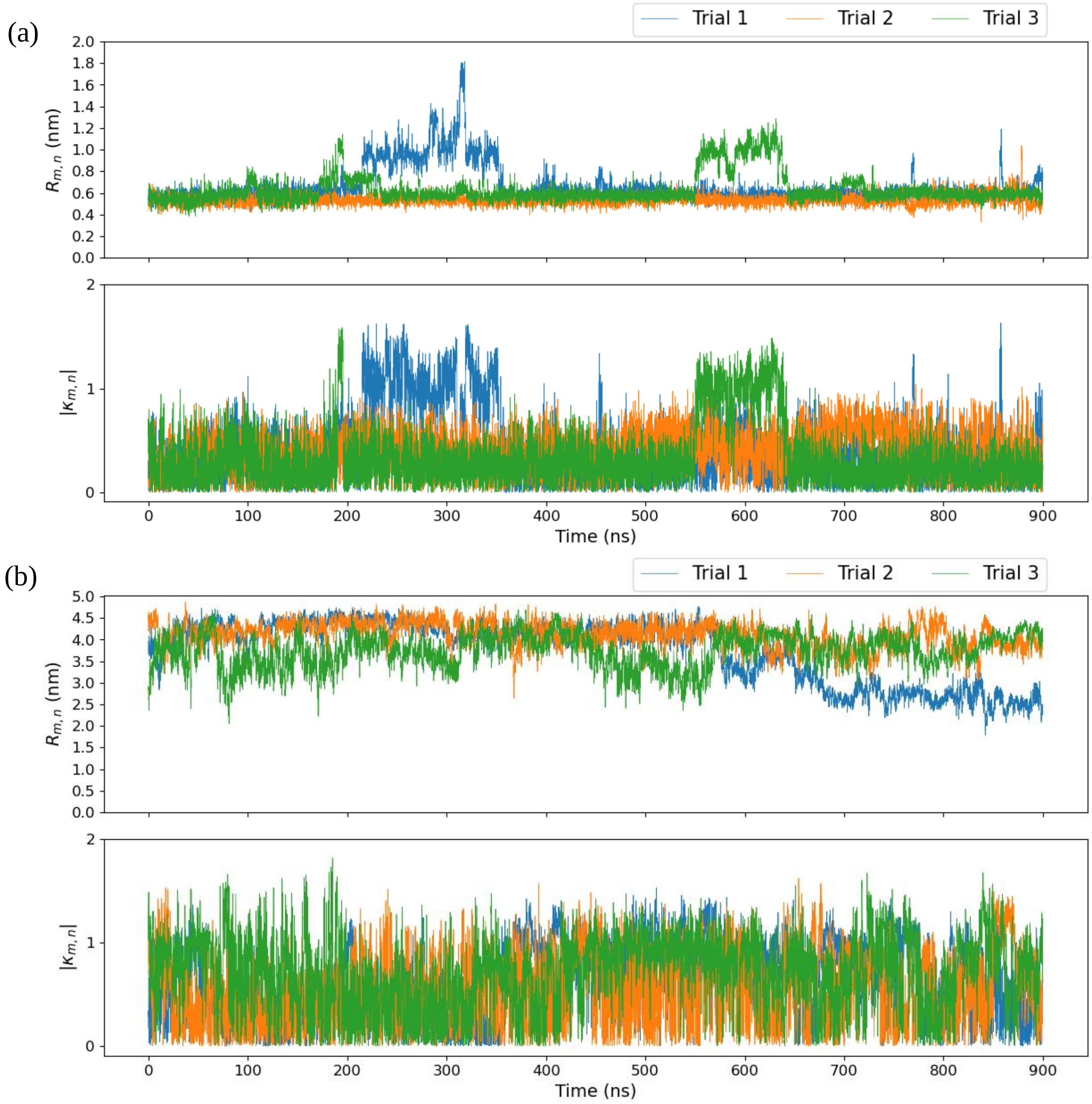
(a)
Trial 1
Trial 2
Trial 3
$R_{m,n}$ (nm)
$|\kappa_{m,n}|$
Time (ns)
(b)
Trial 1
Trial 2
Trial 3
$R_{m,n}$ (nm)
$|\kappa_{m,n}|$
Time (ns)

(c)

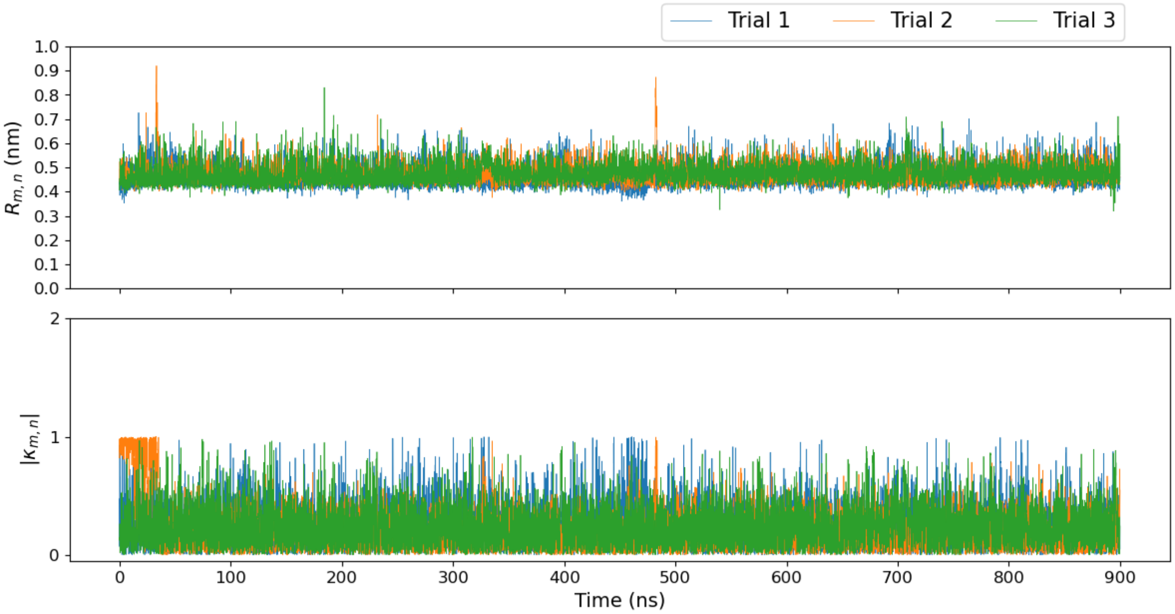
Trial 1
Trial 2
Trial 3
$R_{m,n}$ (nm)
$|\kappa_{m,n}|$
Time (ns)

(d)

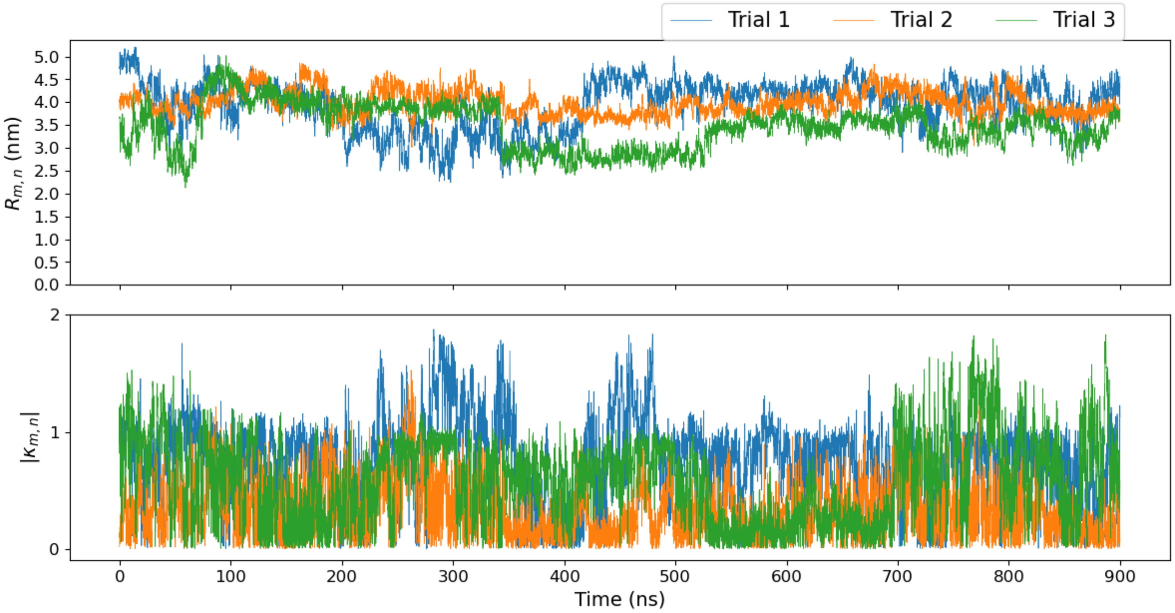
Trial 1
Trial 2
Trial 3
$R_{m,n}$ (nm)
$|\kappa_{m,n}|$
Time (ns)

(e)

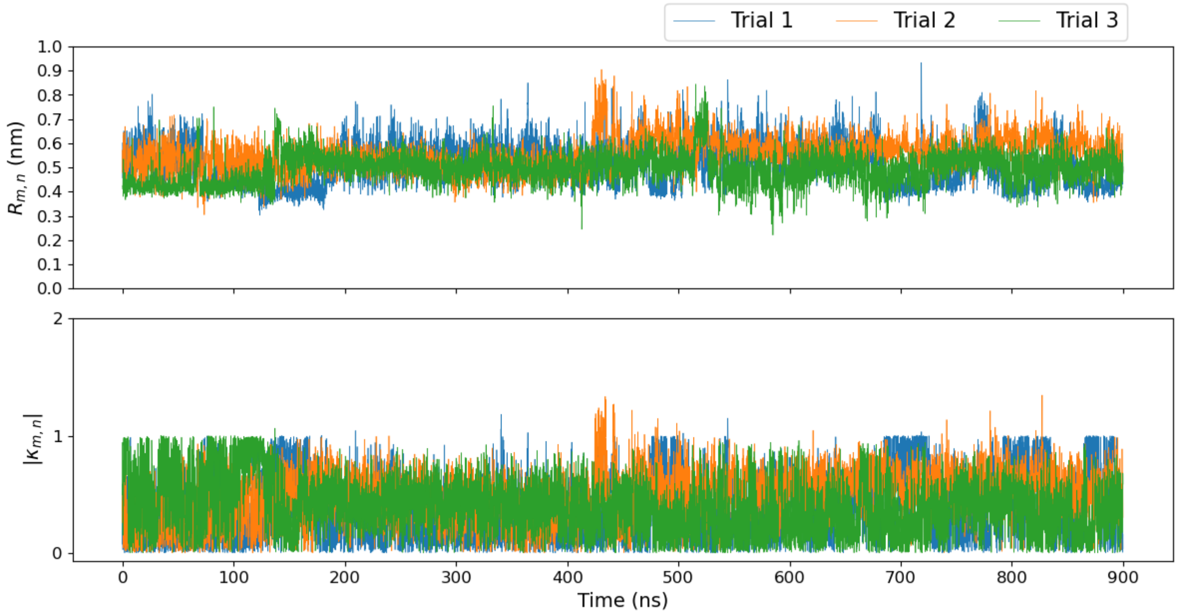

(f)

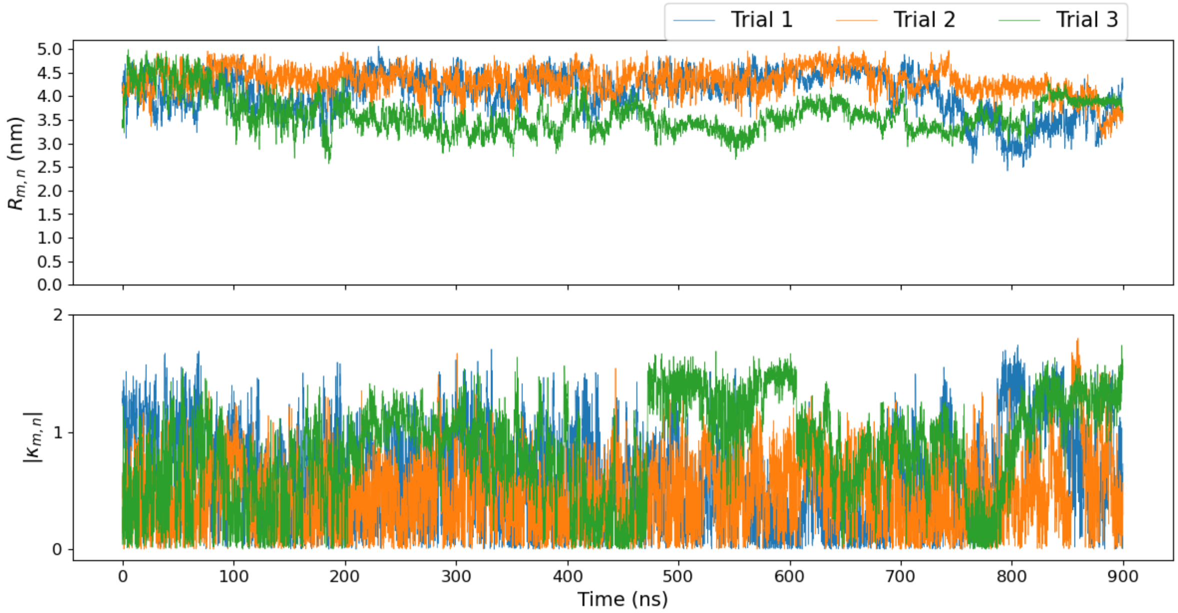

Figure S7. $R_{m,n}$ and $\kappa_{m,n}$ for MD simulation trial 1 (blue), trial 2 (orange), and trial 3 (green) as a function of simulation time for (a) $A_{Cy5}C_{Cy5}$ Iso I, (b) $A_{Cy5}C_{Cy5}$ Iso II, (c) $A_{Cy5.5}C_{Cy5.5}$ Iso I, (d) $A_{Cy5.5}C_{Cy5.5}$ Iso II, (e) $A_{Cy5}C_{Cy5.5}$ Iso I, and (f) $A_{Cy5}C_{Cy5.5}$ Iso II. Each trial consisted of a separate, 1 μs production MD simulation with different starting velocities, and the first 100 ns were taken to be an equilibration period and so were neglected.

(a)

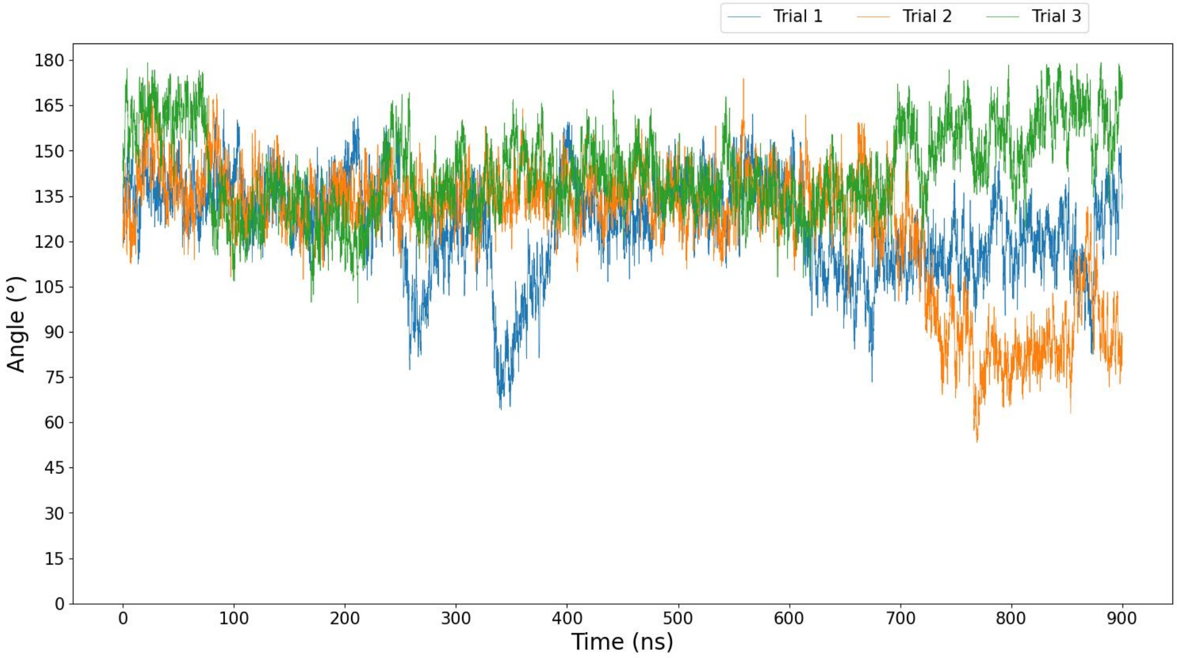

Trial 1
Trial 2
Trial 3
Angle (°)
Time (ns)

(b)

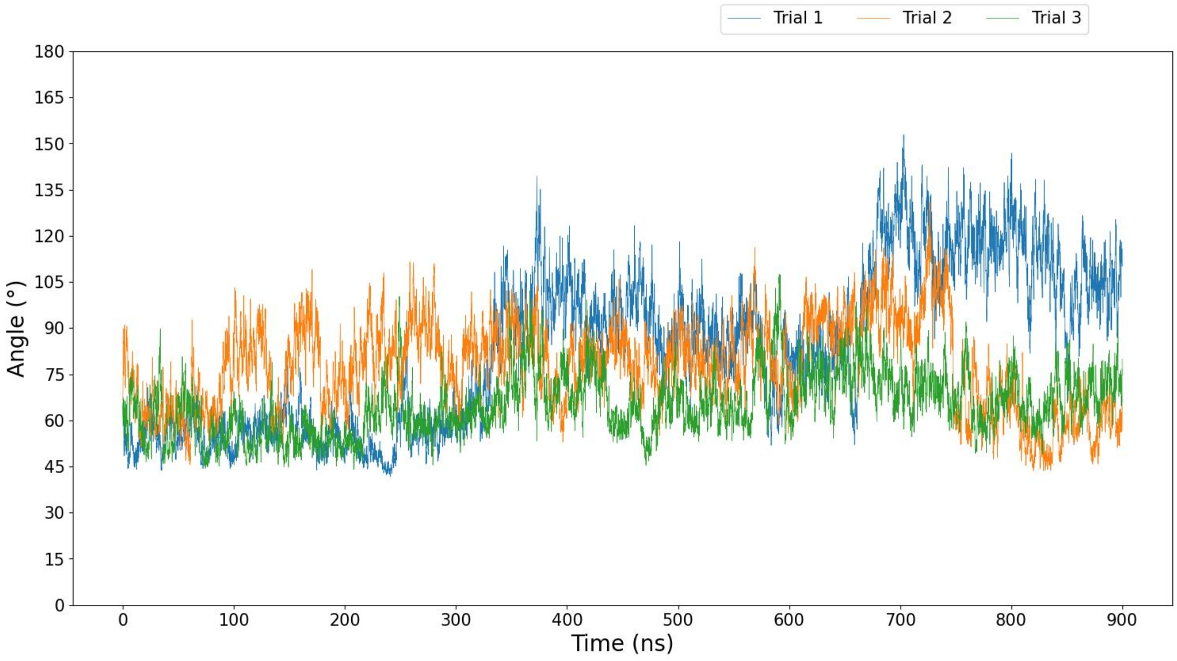

Trial 1
Trial 2
Trial 3
Angle (°)
Time (ns)

(c)

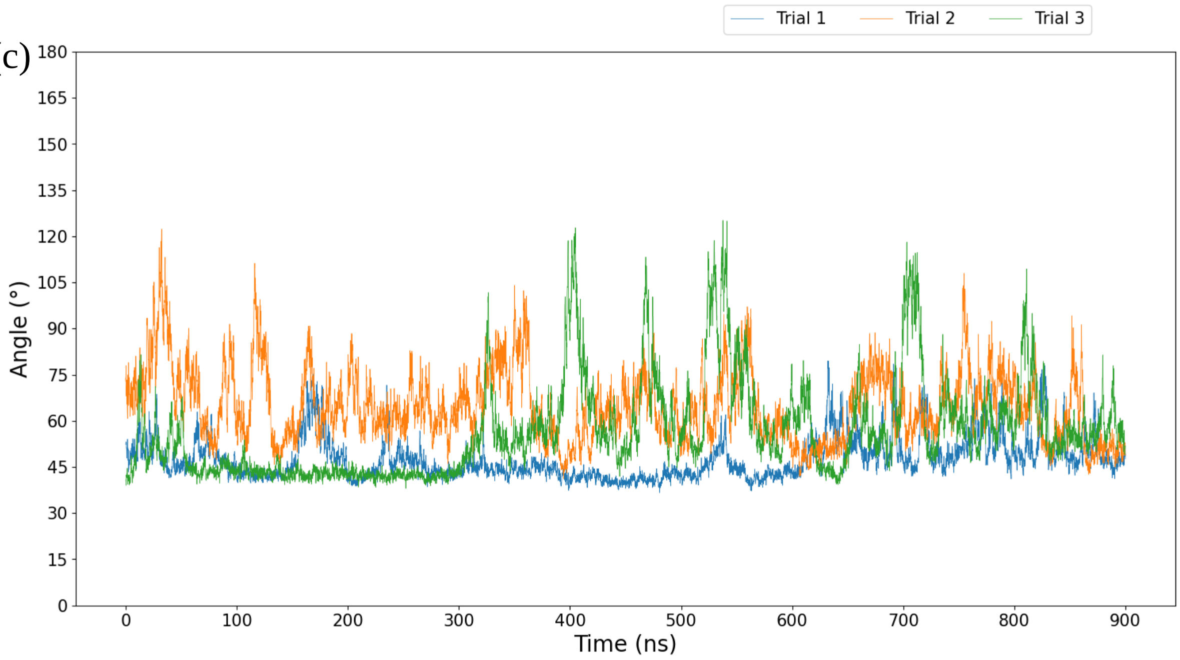
Trial 1
Trial 2
Trial 3
Angle (°)
Time (ns)
0
15
30
45
60
75
90
105
120
135
150
165
180
0
100
200
300
400
500
600
700
800
900

(d)

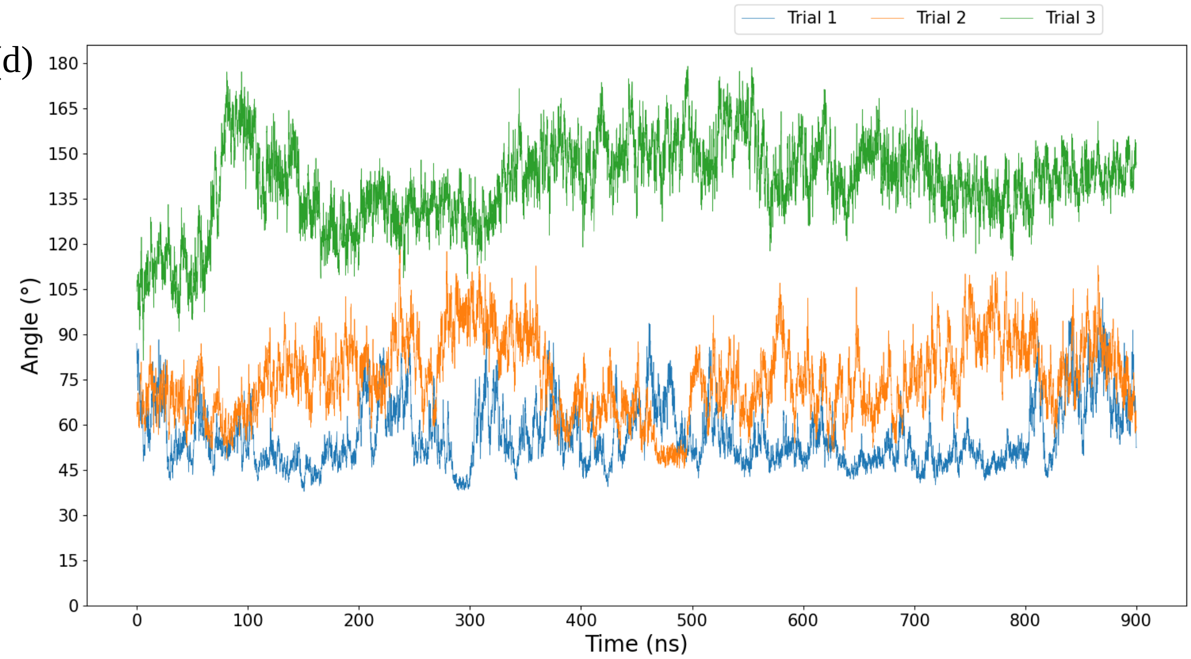
Trial 1
Trial 2
Trial 3
Angle (°)
Time (ns)
0
15
30
45
60
75
90
105
120
135
150
165
180
0
100
200
300
400
500
600
700
800
900

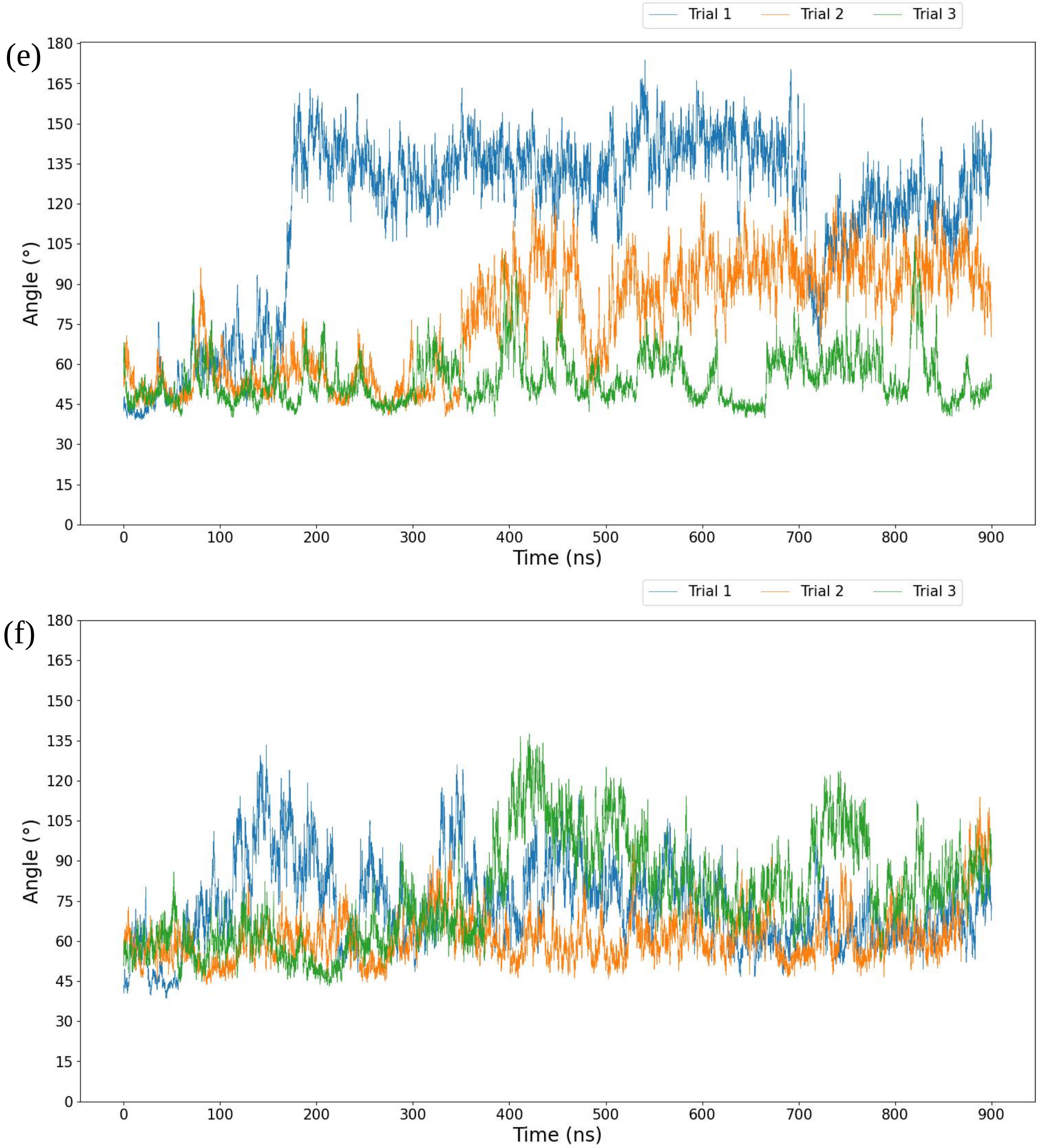

Figure S8. IDA for MD simulation trial 1 (blue), trial 2 (orange), and trial 3 (green) as a function of simulation time for (a) $A_{Cy5}C_{Cy5}$ Iso I, (b) $A_{Cy5}C_{Cy5}$ Iso II, (c) $A_{Cy5.5}C_{Cy5.5}$ Iso I, (d) $A_{Cy5.5}C_{Cy5.5}$ Iso II, (e) $A_{Cy5}C_{Cy5.5}$ Iso I, and (f) $A_{Cy5}C_{Cy5.5}$ Iso II, and the first 100 *ns* were taken to be an equilibration period and so were neglected.

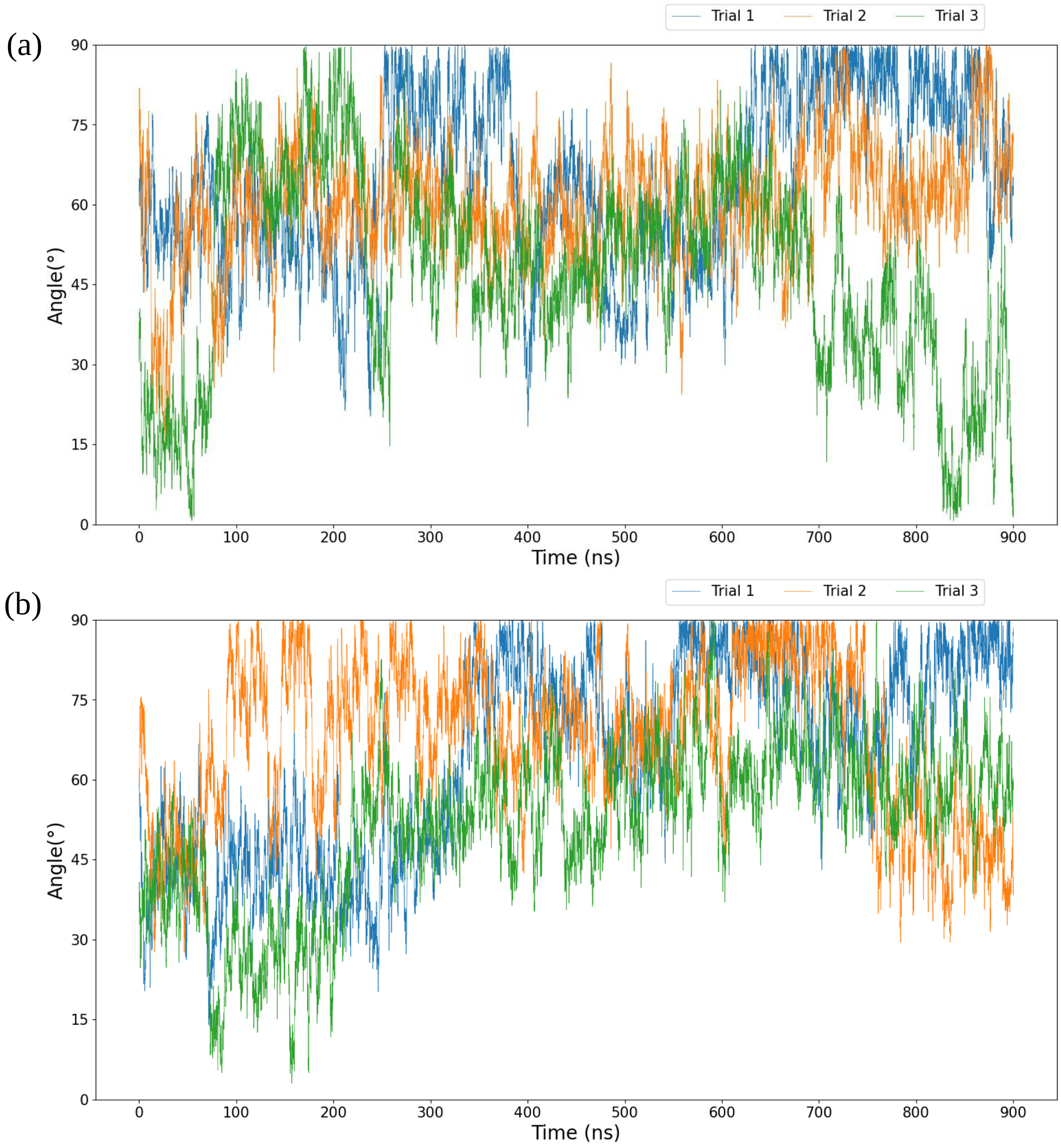
(a)
Trial 1
Trial 2
Trial 3
Angle(°)
Time (ns)
(b)
Trial 1
Trial 2
Trial 3
Angle(°)
Time (ns)

(c)

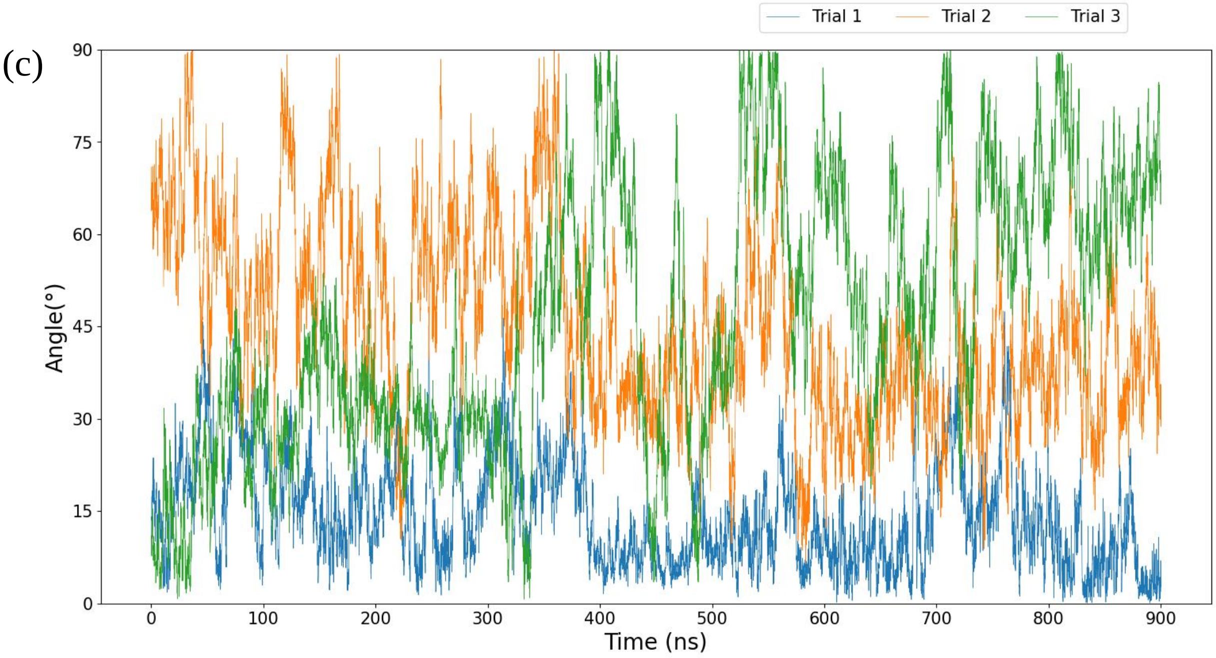
Trial 1
Trial 2
Trial 3
Angle(°)
Time (ns)
0
15
30
45
60
75
90
0
100
200
300
400
500
600
700
800
900

(d)

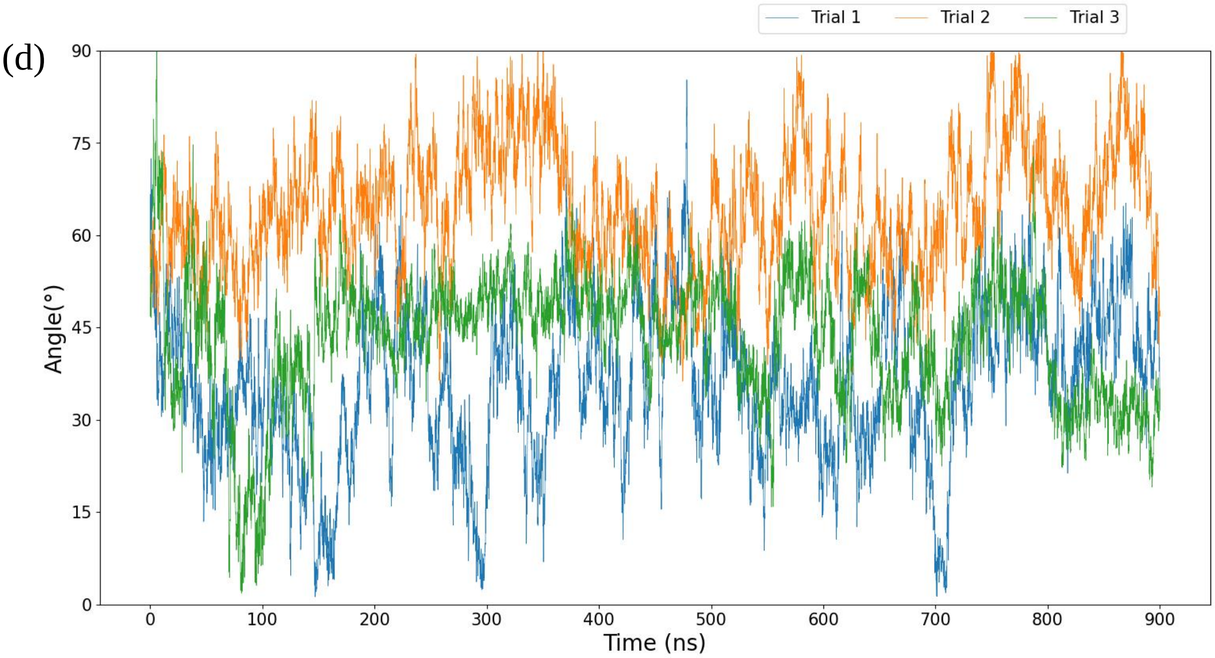
Trial 1
Trial 2
Trial 3
Angle(°)
Time (ns)
0
15
30
45
60
75
90
0
100
200
300
400
500
600
700
800
900

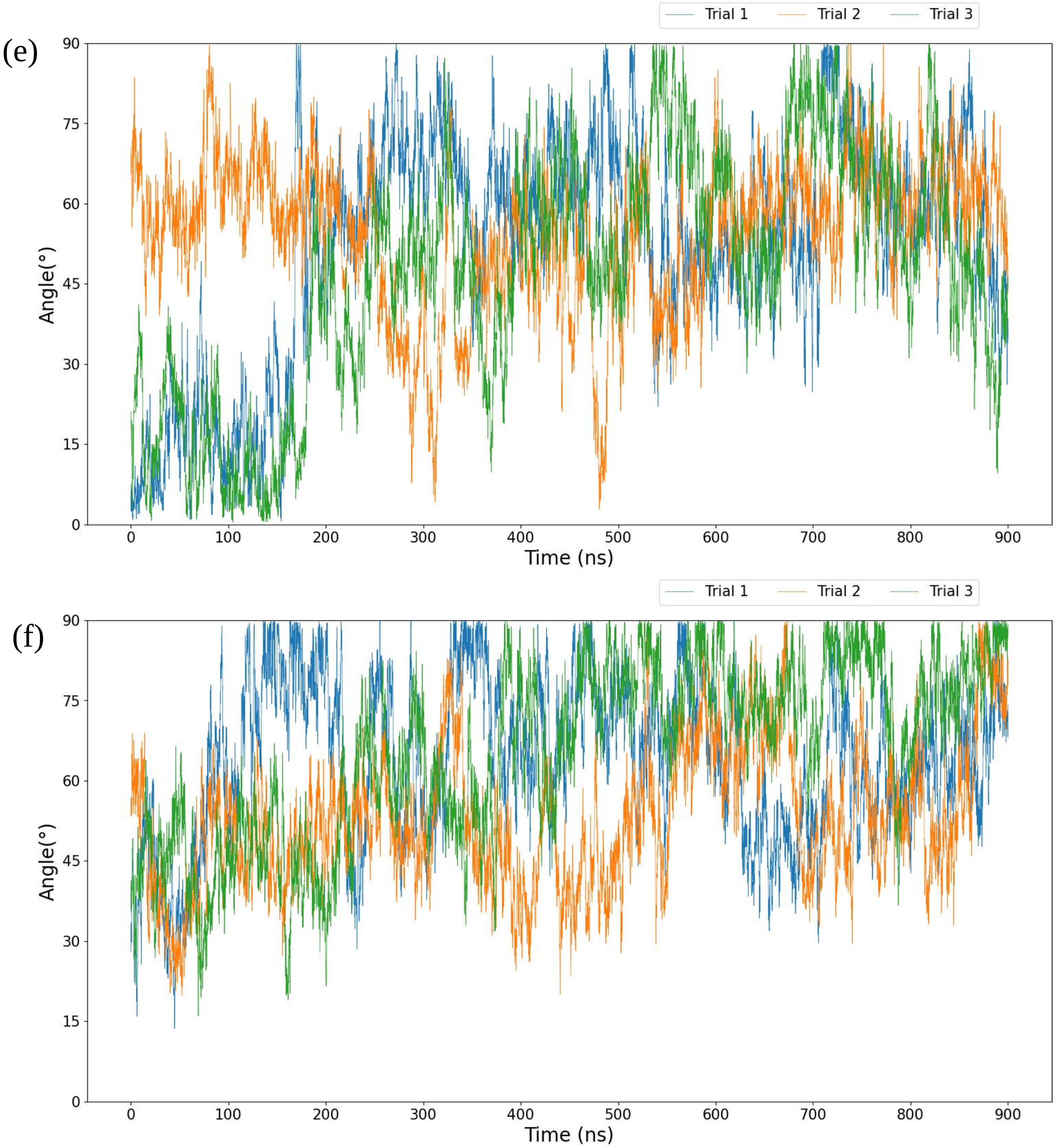

Figure S9. Holliday junction twist angles for MD simulation trial 1 (blue), trial 2 (orange), and trial 3 (green) as a function of simulation time for (a) $A_{Cy5}C_{Cy5}$ Iso I, (b) $A_{Cy5}C_{Cy5}$ Iso II, (c) $A_{Cy5.5}C_{Cy5.5}$ Iso I, (d) $A_{Cy5.5}C_{Cy5.5}$ Iso II, (e) $A_{Cy5}C_{Cy5.5}$ Iso I, and (f) $A_{Cy5}C_{Cy5.5}$ Iso II. Each trial consisted of a separate, 1 μs production MD simulation with different starting velocities, and the first 100 ns were taken to be an equilibration period and so were neglected.

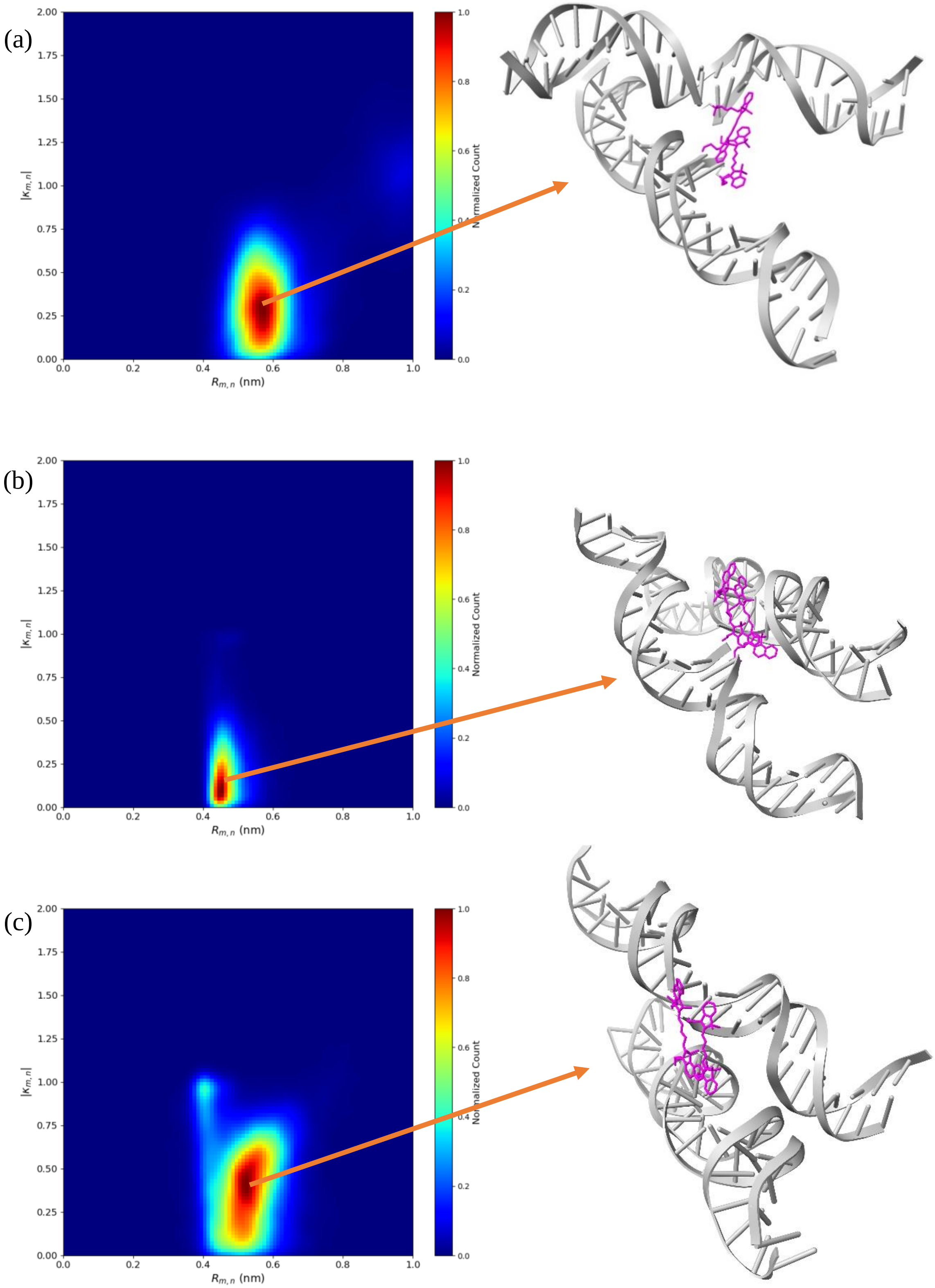

(a)
(b)
(c)
|κm,n|
Rm,n (nm)
Normalized Count

Figure S10. Heatmaps of $|\kappa|$ versus $R_{m,n}$ and corresponding representative snapshots for (a) $A_{Cy5}C_{Cy5}$ Iso I, (b) $A_{Cy5.5}C_{Cy5.5}$ Iso I, and (c) $A_{Cy5}C_{Cy5.5}$ Iso I. The dyes are colored magenta while the DNA is colored gray.

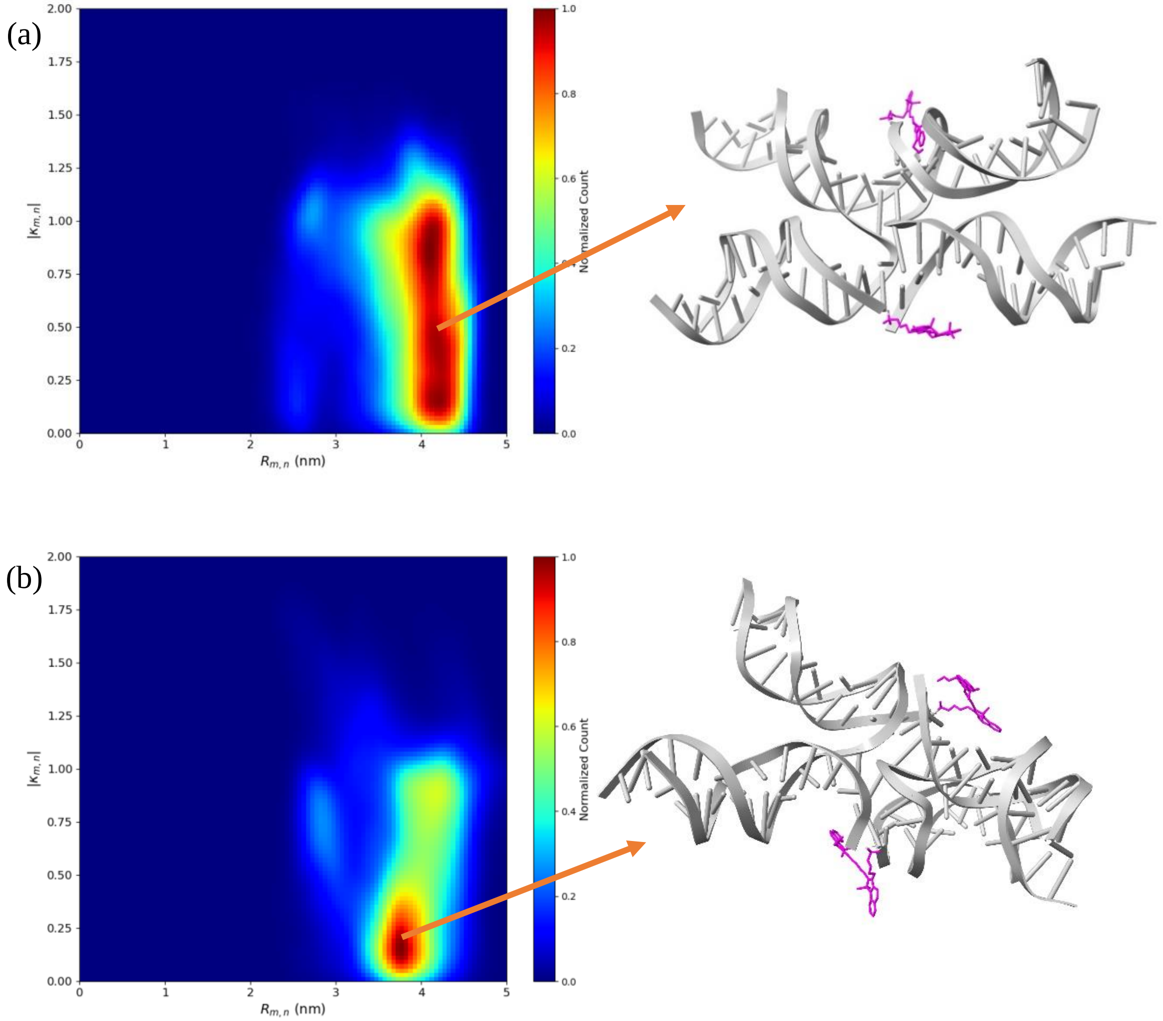

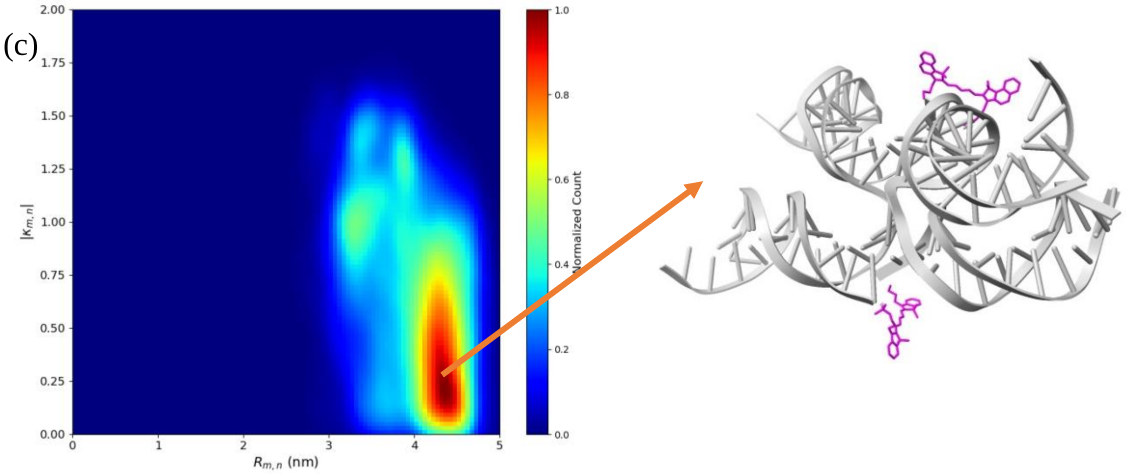

Figure S11. Heatmaps of $|\kappa|$ versus $R_{m,n}$ and corresponding representative snapshots for (a) $A_{Cy5}C_{Cy5}$ Iso II, (b) $A_{Cy5.5}C_{Cy5.5}$ Iso II, and (c) $A_{Cy5}C_{Cy5.5}$ Iso II. The dyes are colored magenta while the DNA is colored gray.

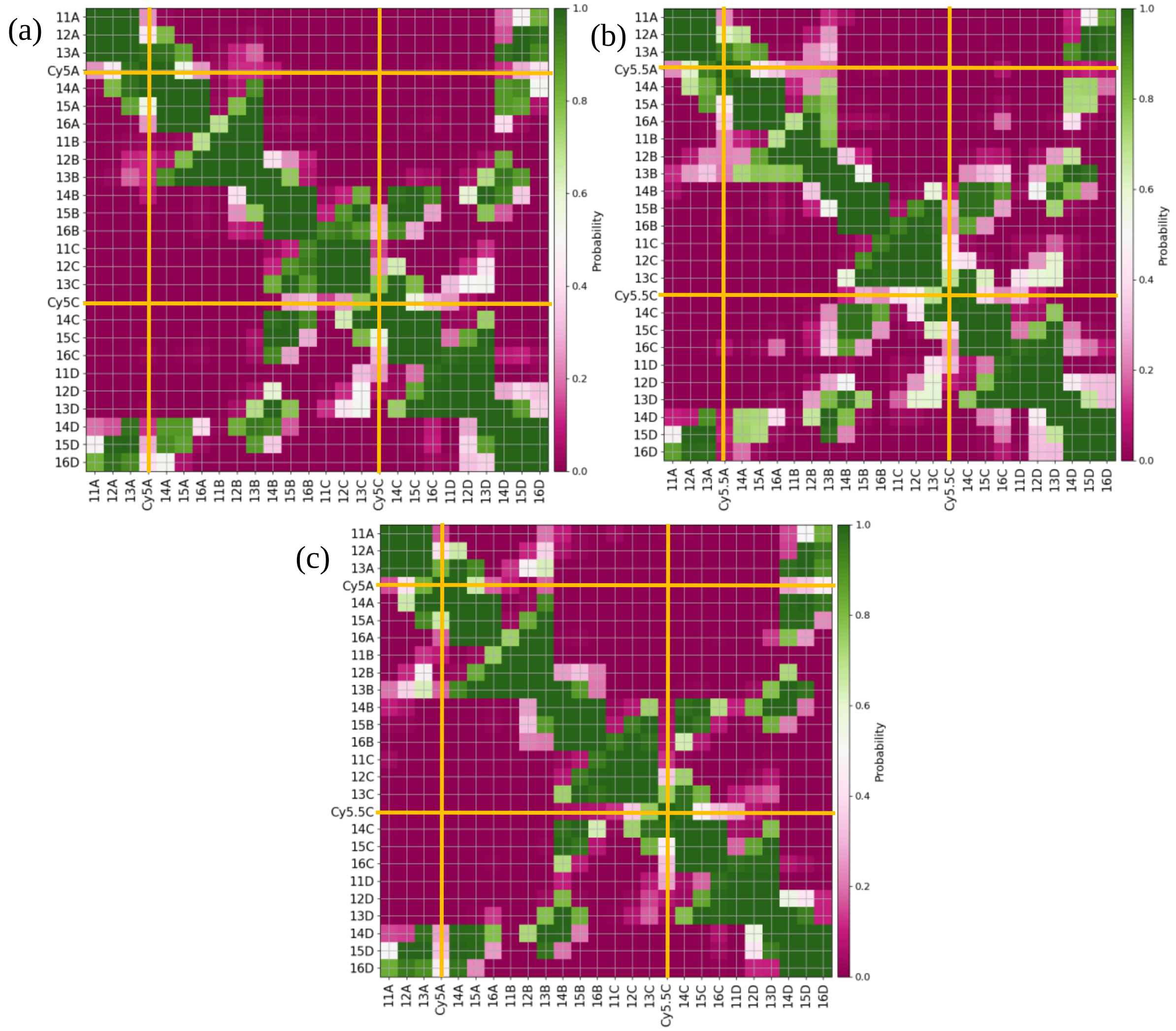

Figure S12. Contact maps for (a) $A_{Cy5}C_{Cy5}$ Iso II, (b) $A_{Cy5.5}C_{Cy5.5}$ Iso II, and (d) $A_{Cy5}C_{Cy5.5}$ Iso II. Dye contacts with all other residues are indicated in the plots with bold orange lines.

Table S1. Average values for $R_{m,n}$, $|\kappa|$, and $|J_{m,n}|$ (when applicable) for the three trials.

System	$R_{m,n}$ trial 1 (nm)	$R_{m,n}$ trial 2 (nm)	$R_{m,n}$ trial 3 (nm)	$\|\kappa\|$ trial 1	$\|\kappa\|$ trial 2	$\|\kappa\|$ trial 3	$\|J_{m,n}\|$ trial 1 (meV)	$\|J_{m,n}\|$ trial 2 (meV)	$\|J_{m,n}\|$ trial 3 (meV)
$A_{Cy5}B_{Cy5}$ Iso I	2.25	1.49	1.69	0.51	1.01	1.07	--	--	--
$A_{Cy5}B_{Cy5}$ Iso II	1.80	2.75	2.70	0.36	0.70	0.83	--	--	--
$A_{Cy5}C_{Cy5}$ Iso I	0.66	0.53	0.64	0.42	0.41	0.34	64.50	87.64	74.80
$A_{Cy5}C_{Cy5}$ Iso II	3.74	4.11	3.74	0.62	0.55	0.71	--	--	--
$A_{Cy5.5}B_{Cy5.5}$ Iso I	1.43	1.18	1.41	1.52	1.09	0.87	--	--	--
$A_{Cy5.5}B_{Cy5.5}$ Iso II	2.29	2.20	1.95	0.31	0.85	0.61	--	--	--
$A_{Cy5.5}C_{Cy5.5}$ Iso I	0.46	0.47	0.47	0.24	0.21	0.24	116.88	107.23	104.58
$A_{Cy5.5}C_{Cy5.5}$ Iso II	3.95	3.99	3.47	0.76	0.34	0.59	--	--	--

$A_{Cy5}B_{Cy5.5}$ Iso I	1.50	1.56	1.06	1.34	1.34	0.36	--	--	--
$A_{Cy5}B_{Cy5.5}$ Iso II	2.67	2.82	2.73	0.59	0.64	0.57	--	--	--
$A_{Cy5}C_{Cy5.5}$ Iso I	0.51	0.56	0.49	0.37	0.49	0.40	104.35	80.2	94.21
$A_{Cy5}C_{Cy5.5}$ Iso II	4.08	4.32	3.60	0.63	0.48	0.85	--	--	--
